# Telomere organization and the interstitial telomeric sites involvement in insects and vertebrates chromosome evolution

**DOI:** 10.1590/1678-4685-GMB-2022-0071

**Published:** 2022-11-14

**Authors:** Marcelo Ricardo Vicari, Daniel Pacheco Bruschi, Diogo Cavalcanti Cabral-de-Mello, Viviane Nogaroto

**Affiliations:** 1 Universidade Estadual de Ponta Grossa, Departamento de Biologia Estrutural, Molecular e Genética, Ponta Grossa, PR, Brazil.; 2 Universidade Federal do Paraná, Centro Politécnico, Programa de Pós-Graduação em Genética, Curitiba, PR, Brazil.; 3 Universidade Estadual Paulista, Instituto de Biociências, Departamento de Biologia Geral e Aplicada, Rio Claro, SP, Brazil.; 4 Universidad de Jaén, Departamento de Biología Experimental, Área de Genética, Jaén, Spain.

**Keywords:** Chromosome rearrangements, fluorescence *in situ* hybridization, heterochromatin, ITS, karyotype evolution

## Abstract

Telomere has a central role in chromosomal stability events. Chromosome ends organized in telomere-loop prevent activation of DNA damage response (DDR) mechanisms, thus keeping the chromosome structure organized. On the other hand, free chromosome ends, dysfunctional telomeres, and interstitial telomeric sequences (ITS) can trigger chromosome rearrangements. Here, the telomere organization, function, and maintenance mechanisms, in addition to ITS types and their involvement in chromosome changes, were revisited. Despite a general (TTAGGG)_n_ sequence being present in vertebrate telomeres, insects show more diversification of their telomere motif. The relation between ITS and chromosome rearrangements was observed in insects and vertebrates, demonstrating different types of genome organization and distribution. Some ITS cannot be considered relicts of chromosome rearrangements because probable they were inserted during a double-strand break repair mechanism. On the other hand, the involvement of telomere sequences participating or triggering chromosome rearrangements or organizing satellite DNA components in several species groups is evident. The genomic assembling advances and applying other methodologies over ITS, and their flanking regions, can help to understand the telomere participation in the chromosomal evolution in species groups with highly diversified karyotypes.

## Telomeres organization and function

### Telomere motifs

**Figure 1 -:**
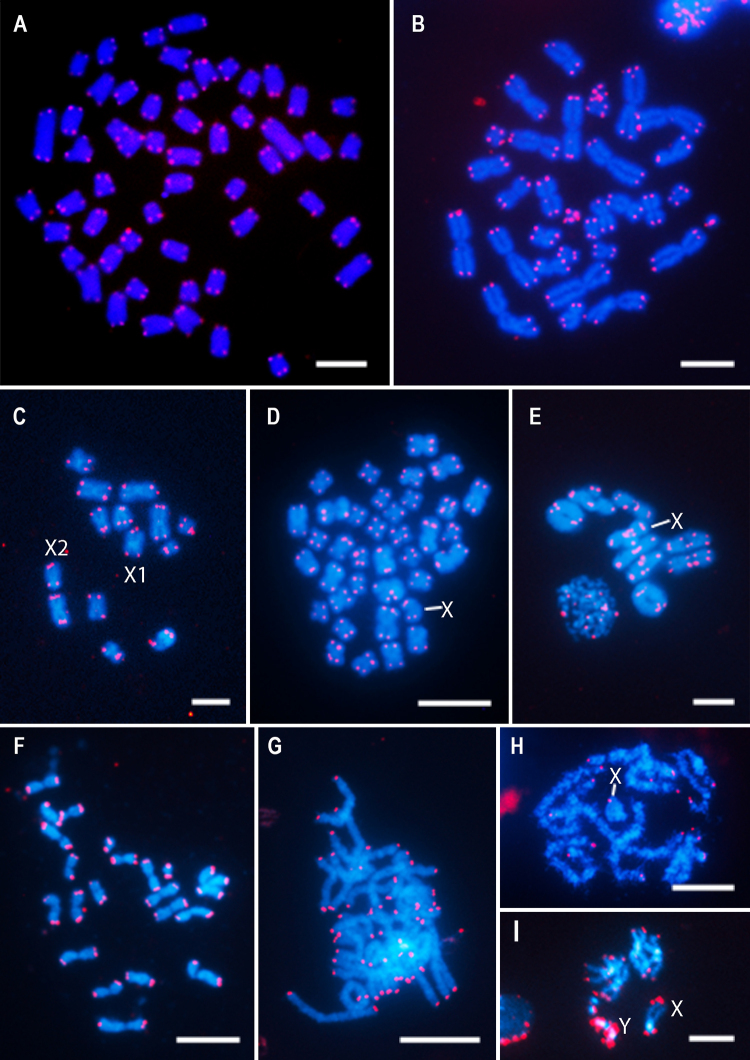
FISH mapping of telomeric repeats (red) in two vertebrates and seven species of insect, belonging to orders Orthoptera, Blattodea, Hemiptera, Hymenoptera, Lepidoptera, Coleoptera, and Diptera. In (A, B) it was used as probe the (TTAGGG)_n_ motif, in (C-I) it was used as probe the “insect” telomere motif (TTAGG)_n_ largely present in multiple groups, and in (G) it was mapped the *HeT*-A retrotransposon. (A) *Apareiodon affinis* fish, mitotic metaphase (2n = 54); (B) *Glossophaga soricina* bat, mitotic metaphase (2n = 32); (C) *Cyclopltiloides americanus*, mitotic metaphase from male embryo (2n = 12AA + X_1_X_2_); (D) *Nauphoeta cinerea*, spermatogonial mitotic metaphase from male (2n = 36AA + X); (E) *Mahanarva spectabillis* male, late diplotene (2n = 9AA + X); (F) *Atta sexdens* female, mitotic metaphase from brain larvae (2n = 22AA); (G) *Helicoverpa armigera* male, pachytene bivalents (n = 31); (H) *Conoderus malleatus* male, pachytene (n = 8 + X); (I) *Drosophila melanogaster* male, mitotic metaphase from brain larvae (2n = 6AA + XY). The chromosomes were counterstained with DAPI (in blue). The sex chromosomes were indicated in some metaphases. Bar = 5 μm.

Telomeres are DNA regions at the end of eukaryotic chromosomes and are essential to their stability and integrity maintenance (Pisano et al., 2008; O’Sullivan and Karlseder, 2010; Galati et al., 2013; Lazzerini-Denchi and Sfeir, 2016). In most organisms, the telomeres consist of tandemly repeated motifs (usually 5 - 8 bp) with telomeric proteins attached to them, capping and protecting the telomeric region (Zakian, 1995). In vertebrates, the hexamer (TTAGGG)_n_ is present (Figure 1A, B), but a considerable variation in the length of the repeated region has been reported (Zakian, 1995).

In other organisms, the size and organization of the telomeric motifs can be distinct. In insects, the presence of the called “insect” telomeric motif (TTAGG)_n_ is common (Figure 1C-F, and H-I), seems to be ancestral, and it is also shared by other arthropods (Frydrychová et al., 2004; Vítková et al., 2005). Although the structure of the telomeric motif could be quite variable within several insect groups because it can be lost or replaced by alternative mechanisms for maintaining telomere, making the study of the telomere structure and evolution of this class of animals interesting. Examples of the occurrence of non-canonical “insect” telomeric motif are observed in Coleoptera (beetles), with the description of the motif (TCAGG)_n_ on chromosome termini in representatives of Tenebrionidae (Mravinac et al., 2011) and Cleridae (Prušáková et al. 2021), and the recently discovered motif (TTGGG)_n_ on the Geotrupidae *Anoplotrupes stercorosus* (Prušáková *et al.*, 2021). Remarkably among Diptera, the transposition of the non-long terminal repeat (non-LTR) retrotransposon was co-opted to maintain the telomere of *Drosophila* (Mason and Biessmann, 1995; Pardue and DeBaryshe, 2003; Figure 1G), and in basal dipterans, as chironomid midges, the recombination of long terminal repeats (LTR), i.e., satellite DNAs, maintains the telomeres (Nielsen and Edstrom, 1993). 

Interestingly, in *Bombyx mori* (Lepidoptera; Fujiwara et al., 2005), *Myzus persicae* (Hemiptera; Monti et al., 2013), *Pediculus humanus* (Phthiraptera), and *Tribolium castaneum* (Coleoptera; Osanai et al., 2006), the (TTAGG)_n_ is interspersed with inserted non-LTR retrotransposable elements. This condition could be putatively an intermediate state between the canonical insect telomere and retrotransposon-based ones (Mason et al., 2016). In the honeybee (*Apis mellifera*, Hymenoptera), the telomere is also exceptionally non-canonical, forming a mosaic composed of TTAGG interspersed with TCAGGCTGGG, TCAGGCTGGGTTGGG, and TCAGGCTGGGTGAGGATGGG (Garavís et al., 2013). Finally, in some other groups of insects, the (TTAGG)_n_ is facultatively present, as in Hemiptera, Odonata, Hymenoptera, Neuroptera, and Coleoptera. While, in other few orders, including Ephemeroptera, Dermaptera, Raphidioptera, Siphonaptera, and Mecoptera, until now, the (TTAGG)_n_ was not evidenced, but no alternative telomeric motif was detected, deserving additional investigation, as just a few species were investigated (revised by Kuznetsova et al., 2020).

Based on genomic data, i.e., chromosome-level assemblies of 180 species of insects belonging to 8 orders, Lukhtanov (2022) added important data about the diversity of telomeres in this group. He confirmed some previous information about the insect-telomere structure and noticed new variations. In general, it was observed: *(i)* short repeats (the canonical telomeres); *(ii)* mononucleotide telomeres that consist of a long array of (A)_n_ and (T)_n_ at 5’and 3’ends, respectively; *(iii)* main short repeats with variants of short repeats; *(iv)* main short repeats interspersed with telomere-specific non-LTR retrotransposon (*TRAS*, *SART* families or both); *(v)* long repeats; *(vi)* long repeats in one telomere and arrays of short repeats (TTAGGTCTGGG)_n_ at the other end; *(vii)* non-LTR retrotransposons, including the *HeT-A*, *TAHRE*, and *TART* families (Lukhtanov, 2022).

### Telomere organization

In most cases, the telomere organization has the 5’ cytosine (C)-rich at the end of one strand, while the 3’ strand end is guanine (G)-rich (Lazzerini-Denchi and Sfeir, 2016; Aksenova and Mirkin, 2019). The G-rich single-strand results from the inability of DNA polymerase to replicate chromosome ends (Watson, 1972; Lazzerini-Denchi and Sfeir, 2016). DNA replication requires a primer containing a free 3’ -OH group to start the DNA synthesis (Watson, 1972; Olovnikov, 1973). During the replication (which must occur in the 5’→3’ direction), the telomeres generated by continuous strand synthesis have blunt ends or small 5’ protrusions (Lazzerini-Denchi and Sfeir, 2016). The end of the discontinuous strand has a 3’ single-strand end, which comprises the segment from the removal of the RNA primer, referring to the beginning of the Okazaki fragment (Lazzerini-Denchi and Sfeir, 2016). The DNA polymerase inability to synthesize the end of chromosomes is named the end replication problem (Watson, 1972; Olovnikov, 1973). 

According to the end replication problem mechanism, each discontinuous strand replication event leads to an 8-12 bp gap on the 3’ termini, resulting in a DNA shortening at each cell cycle (Makarov et al., 1997; Blackburn et al., 2006). The telomerase enzyme repairs the chromosome ends length in some specific cells by adding telomeric repeats in the 3’ G-rich strand as an important mechanism of damage prevention (Makarov *et al.*, 1997; Blackburn *et al.*, 2006). In addition to its participation in telomerase action, the G-rich strand end has an essential role in telomere organization. This strand is prone to form stable secondary structures, including quadruplex DNA (G4-DNA structures) (Sundquist and Klug, 1989; Williamson et al., 1989) that impair the replication machinery as it progresses through telomeric DNA (Lazzerini-Denchi and Sfeir, 2016). In this way, G4-DNA protects the chromosome ends and inhibits the telomerase action (Zahler et al., 1991; Smith et al., 2011).

**Figure 2 -:**
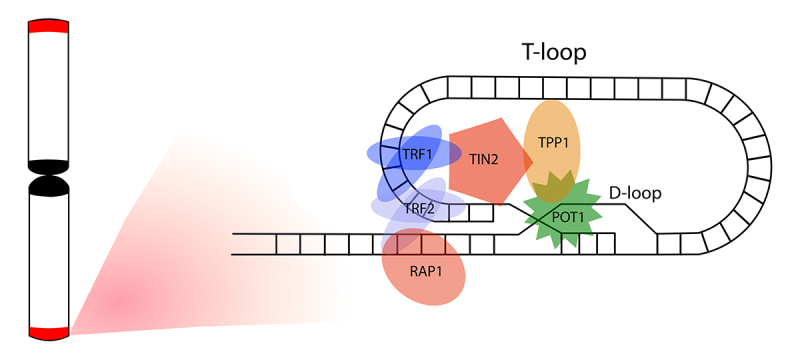
Schematic representation of the T-loop organization. The telomere DNA loops back on itself, forming the T-loop. The 3’ G strand extension invades the duplex telomeric repeats and forms a D-loop. During the organization, the telomeric DNA is bound by the specialized shelterin complex and packaged into a t-loop configuration. The shelterin complex is organized by TRF1, TRF2, TIN2, RAP1, TPP1, and POT1. The six-subunit proteins protect the chromosome ends from the DNA damage signaling pathway and DNA repair mechanisms.

Besides the G4-DNA structures, the telomeres can also organize a structure called a telomere loop (t-loop) (Griffith et al., 1999). Telomeres end with a single-stranded G-rich overhang that can invade the preceding double-stranded region to generate a particular lariat-like structure, the t-loop (Griffith *et al.*, 1999; Figure 2). In the t-loop generation, the G-rich single-strand extension invades a precedent segment containing the duplex telomeric repeats and forms a displacement loop (d-loop) (Griffith *et al.*, 1999; Figure 2). The d-loop binds telomeric proteins capping the chromosome ends (van Steensel et al., 1998; de Lange, 2005). The proper telomere capping depends on the interaction of some proteins with telomeric repeats, called the shelterin complex (Pisano et al., 2008; Galati et al., 2013; Lazzerini-Denchi and Sfeir, 2016).

### Telomeric proteins complex 

Shelterin organizes a specialized multiprotein complex on telomeric region composed of six distinct proteins: telomere repeat-binding factor 1 (TRF1), telomere repeat-binding factor 2 (TRF2), protection of telomere 1 (POT1), telomere protection 1 (TPP1), TRF1-interacting nuclear factor 2 (TIN2), and repressor activator protein 1 (RAP1) in vertebrates (Pisano et al., 2008; Galati et al., 2013; Lazzerini-Denchi and Sfeir, 2016; Figure 2), besides variants forms presented in some organisms. The shelterin complex is essential to telomere length maintenance and protects the chromosome ends (Ichikawa et al., 2015; Lazzerini-Denchi and Sfeir, 2016). TRFs 1 and 2 bind to the double-stranded DNA of telomeric sequences and organize the core of the shelterin complex (Court et al., 2005; Lazzerini-Denchi and Sfeir, 2016). In the t-loop organization, TRF1 attaches to an internal nucleosome site and induces the mobility and condensation of the telomeric DNA (Pisano *et al.*, 2010; Ichikawa *et al.*, 2015). The TRF2 protein also participates in the telomeric chromatin structure, reducing nucleosome density and increasing the spacing among telomeric nucleosomes (Ichikawa *et al.*, 2015). In addition, TRF2 plays an important role as a t-loop facilitator, and its loss leads to an increase in DNA damage response (DDR) pathways, chromosomal end-fusions, and cell senescence (van Steensel et al., 1998; de Lange, 2005; Fouché et al., 2006). POT1 is the third DNA-binding component within shelterin (Wu et al., 2010; Lazzerini-Denchi and Sfeir, 2016). Telomeres recruits POT1 by interacting with TPP1 and coat the single-stranded part of the TTAGGG repeats with its oligonucleotide/oligosaccharide binding folds (Wu *et al.*, 2010; Lazzerini-Denchi and Sfeir, 2016). TIN2 and RAP1 do not bind directly to telomeric repeats, although they interact with other shelterin in this region (Wu *et al.*, 2010; Lazzerini-Denchi and Sfeir, 2016). In addition to shelterin, another protein complex called CST-complex (composed by Ctc1, Stn1, and Ten1 in humans) binds to telomeric G-rich single-stranded promoting telomere protection and telomerase recruitment (Gao et al., 2007; Rice and Skordalakes, 2016).

### Telomeric RNA 

Although telomeres are highly condensed and heterochromatic, they show a dynamic chromatin structure as they are considered transcriptionally active (Azzalin et al., 2007; Xu et al., 2010). In addition to telomeric repeats and shelterin, telomeres are also made up of non-coding RNA molecules of the type (UUAGGG)_n_, called Telomeric Repeat-containing RNA (TERRA) (Azzalin *et al.*, 2007; Xu *et al.*, 2010). TERRA is transcribed from the C-rich telomere strand, interacts with some telomeric proteins, participates in the transitional states of euchromatin and heterochromatin, and regulates telomerase activity (Azzalin *et al.*, 2007; Xu *et al.*, 2010).

TERRA can also form stable DNA/RNA hybrids with the C-rich telomeric strand, thus resulting in the displacement of the G strand, giving rise to an R-loop structure (Chawla and Azzalin, 2008; Santos-Pereira and Aguilera, 2015). R-loops prevent DNA replication progress by causing replication-fork stalling, collapse, and double-strand breaks (DSBs) (Gómez-González et al., 2011; Balk et al., 2013). Studies also suggest that TERRA is involved in telomere heterochromatin formation (Deng et al., 2009; Maicher et al., 2014).

### Function

Telomeres protect the chromosome ends against inappropriate recombination, exonuclease attacks, and oxidative damage, thus maintaining the integrity and stability of the chromosome (de Lange, 2002). Therefore, the telomeres avoid chromosome ends recognition as DSBs by the DNA repair machinery (Lazzerini-Denchi and Sfeir, 2016; Slijepcevic, 2016). At the same time, telomeres allow the correct anchoring of the chromosomes to the nuclear membrane, ensure the three-dimensional structure of the nucleus and the proper spatial distribution of the chromosomes during cell proliferation (Zakian, 1995; Luderus et al., 1996). On the other hand, the organization of a dysfunctional telomere generates unstable DNA sites, which behave as DSB regions, triggering chromosomal rearrangements (Perry et al., 2004; Slijepcevic, 2016; Bolzán, 2017).

X-rays were used as inducers of chromosomal aberrations and demonstrated that broken chromosomes usually fused with their sister chromatids, generating a Breakage Fusion-Bridge mechanism (McClintock, 1941, 1987). In this mechanism, the terminal regions at the fusion sites were always lost, thus evidencing that the broken chromosomes (without the intact terminal protective “caps” on telomeres, or t-loop) were subject to fusion events (McClintock, 1987). The McClintock studies were important landmarks in chromosomal instability proposal associated with chromosome ends. Interestingly, after irradiation and breaks the end of chromosomes can be healed by de novo telomere addition, as documented in some organisms, including the homopteran insect *Planococcus lilacinus* (Mohan et al., 2011).

## Telomere length maintenance mechanisms

In each DNA replication round, naturally, telomeres lose a segment of their repetitive sequence (Watson, 1972). In the absence of mechanisms that prevent telomere shortening during cell proliferation, there would eventually be an excessive decrease in the terminal region and the activation of a DDR mechanism, leading to cellular senescence (de Lange, 2005). It is known that telomeric DNA has difficulties to replicates due to their repetitive organization, its ability to form secondary structures, as well as the presence of the shelterin complex (Sfeir et al., 2009; Bah et al., 2011; Lopes et al., 2011; Paeschke et al., 2011; Anand et al., 2012). Telomeric replication requires G4-DNA structures to relax and disassemble the t-loops, which demand specialized enzymes, such as several DNA helicases (Croteau et al., 2014; Vannier et al., 2014; Geronimo and Zakian, 2016; Mendoza et al., 2016; Poole and Cortez, 2016). 

Telomeric sequences are added to chromosome ends in specific tissues by the telomerase enzyme action (Kolquist et al., 1998). Telomerase acts as an RNA-dependent DNA polymerase, a type of reverse transcriptase that uses an intrinsic RNA template to transcribe telomeric repeats at chromosome ends, avoiding telomere shortening (Greider, 1995). Telomerase has a catalytic subunit ribonucleoprotein complex called Telomerase Reverse Transcriptase (TERT) and an RNA template, the Telomeric RNA Component (TERC) (Chen and Lingner, 2013). The function occurs by adding telomeric repeats to the G-rich single-stranded end from the reverse transcription of the telomerase RNA template into DNA (Greider, 1995). The activity is controlled by CST-complex (Chen and Lingner, 2013), which after the G-rich strand extension, displaces telomerase, remove secondary structures, and recruit the DNA polymerase α/primase complex to synthesize the C-rich strand (Diede and Gottschling, 1999; Qi and Zakian, 2000; Aksenova and Mirkin, 2019). Thus, the absence of telomerase activity in somatic cells leads to a decrease in telomeric repeat number in each cell cycle, promoting cell senescence (Kolquist *et al.*, 1998; Bolzán et al., 2000; Hines et al., 2005). 

Interestingly, in addition to maintaining the length of telomeres, telomerase can catalyze telomeric repeats synthesis to non-telomeric sites on chromosomes (Melek and Shippen, 1996; Aksenova and Mirkin, 2019). Thus, the telomerase repair activity can lead to chromosome instability and fragmentation when competing with DNA repair machinery on broken ends (Slijepcevic, 1998, 2016). Some proteins binding in the lesion point could prevent the telomerase attachment to DSBs (Slijepcevic and Al-Wahiby, 2005). As mentioned above, dysfunctional telomeres or DSBs appear to be repaired by DNA machinery, sometimes with telomere and telomerase action in chromosome rearrangements.

## DNA repair mechanisms 

Free chromosomal ends appear as DSBs and can be targeted for DNA repair if not adequately protected from the DDR machinery. The DNA lesion repair could occur through many pathways, such as Non-homologous End Joining (NHEJ); Microhomology-mediated End Joining (MHEJ), also called alternative NHEJ; Homologous Recombination (HR); Break-induced DNA Replication (BIR); and Single Strand Annealing (SSA) (Heyer, 2015; Ceccaldi et al., 2016; Lazzerini-Denchi and Sfeir, 2016; Rodgers and McVey, 2016; Kramara et al., 2018; Seol et al., 2018). Sometimes, the DNA repair mechanisms can cause chromosomal rearrangements, leading to unequal distribution of genetic material to daughter cells, thus evidencing the importance of an intact telomeric region during cell division (Slijepcevic, 1998, 2016; Bolzán, 2017; Aksenova and Mirkin, 2019).

**Figure 3 -:**
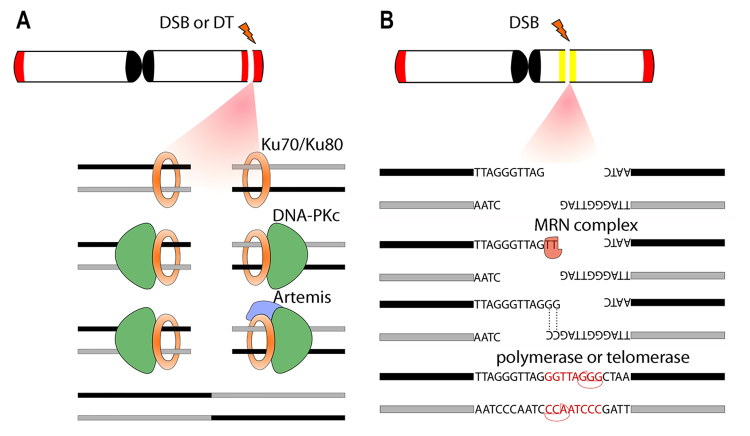
Schematic representation of Non-homologous end joining mechanisms (NHEJ). In (A) classical-NHEJ (c-NHEJ), a free chromosome end generated by double-strand breaks (DSB) or dysfunctional telomere (DT) could be repaired with minimal sequence alterations. The mechanism is initiated with the Ku70/Ku80 heterodimer binding to free chromosome ends. Ku proteins recruit DNA-PKcs to promote phosphorylation. After, the terminal end-processing enzyme Artemis cleaves single-stranded overhangs, then DNA ligase 4 (LIG4) and the scaffold protein XRCC4 connect the free ends. In (B), the alternative-NHEJ (alt-NHEJ) can generate extensive nucleotide deletions or insertions during the DNA repair process. A series of proteins act in alt-NHEJ ends resection, among them poly(ADP-ribose) polymerase 1 (PARP1), MRN complex (MRE11-RAD50-NBS1), and CtBP-interacting protein (CtIP). After ends resection on lesion point, DNA polymerase θ (Pol θ) is recruited to promote end joining. In alt-NHEJ, sometimes in a differential way, the telomerase enzyme is proposed to perform telomere motifs addition to terminal ends after ends resection, thus generating short-ITS.

**Figure 4 -:**
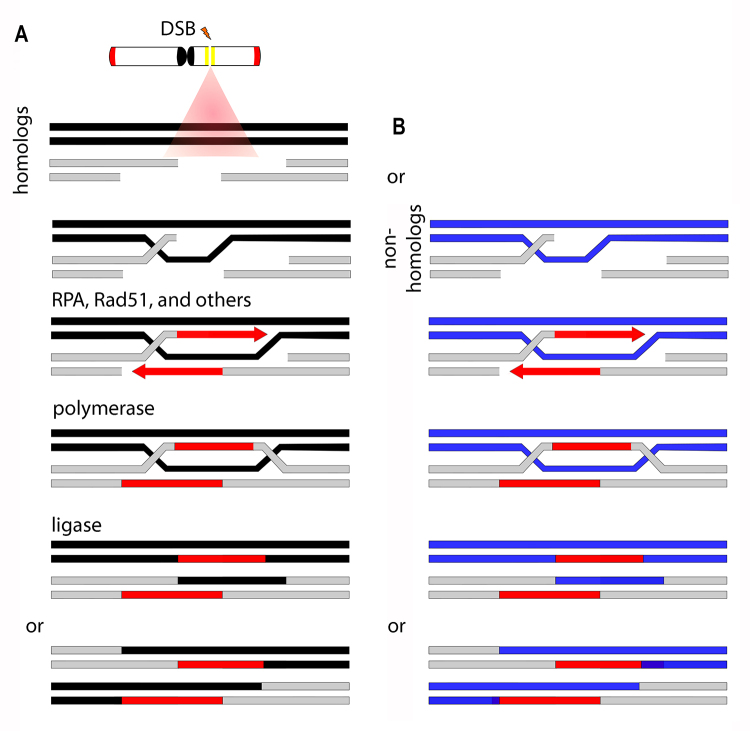
Homologous recombination repair is schematized in (A). After a DSB, the newly released DNA ends are processed to produce long stretches of 3’-terminal single-stranded DNA (ssDNA). Replication Protein A (RPA) binds to ssDNA ends, avoiding potential secondary structure formation and protecting the ssDNA degradation by nucleases. The RPA is replaced with Rad51 mediator help, and the ATP-dependent DNA-binding protein extends the strand at the end of the DNA, organizing the pre-homologous complex. The pre-homologous complex is responsible for finding a double-stranded DNA sequence (dsDNA). The 3’ end of presynaptic ssDNA can initiate the duplication using homologous dsDNA as a template. From this point, the mechanism can be directed to several different molecules that restore DNA. Sometimes, the repair mechanism could find the same DNA repeat region of the DSB point in a non-homologous chromosome (B) and use this dsDNA as a template called non-homologous recombination or non-allelic homologous recombination.

NHEJ is the main DSB repair pathway in the cells (Lazzerini-Denchi and Sfeir, 2016). The mechanism receives this name because, during a DSB, the damaged region of DNA loses some nucleotides, generating non-complementary single-stranded ends subject to a complex repair mechanism (Figure 3). Thus, unlike the HR mechanism, the NHEJ does not require DNA strand homology to guide repair. There are two NHEJ pathways, classical NHEJ and alternative NHEJ (revised in Lazzerini-Denchi and Sfeir, 2016). Classical NHEJ (Figure 3A) repairs DSBs with minimal changes in the sequence, while alternative NHEJ (Figure 3B) can generate large segments possessing deletion or insertion (Lazzerini-Denchi and Sfeir, 2016). During the alternative NHEJ, a probable involvement of telomerase inserting telomeric motifs (Figure 3B) to perform DSB repair was verified, generating short ITS (Ruiz-Herrera et al., 2008; Lazzerini-Denchi and Sfeir, 2016). SSA has a similar alternative NHEJ mechanism and involves the annealing of homologous repeat sequences that flank a DSB, which causes a deletion rearrangement between the repeats (for a review, see Bhargava et al., 2016). On the other hand, the HR mechanism (Figure 4) has action in repairing DSBs without rescuing paralyzed or collapsed replication forks in chromosomal rearrangements, horizontal gene transfer, and meiosis (Pierce et al., 2001). Sometimes, this DNA repair pathway could occur between two lengths of DNA that have high sequence similarity but are not alleles in a mechanism called non-homologous recombination or non-allelic homologous recombination (Parks et al., 2015). This mechanism could promote a concerted evolution of the repeat units and is a common mechanism for generating genome rearrangements (Parks *et al.*, 2015; Barros et al., 2017; Glugoski et al., 2018).

The Breakage-Induced-Replication (BIR), or recombination-dependent DNA replication, is a repair mechanism usually triggered when a single-stranded break in DNA occurs (Anand et al., 2013). Phosphodiester bond break in the polynucleotide strand is the primary type of spontaneous DNA damage. When the DNA duplication fork encounters one of these breaks, the single-stranded end formed needs to be repaired by HR. These breaks can also be detected at the chromosome ends in telomerase-deficient cells and trigger a BIR repair (Anand *et al.*, 2013). The BIR mechanism begins with the invasion of the single-stranded to a homologous DNA sequence, which uses it as a template to replicate until the next duplication fork or the chromosome end (Anand *et al.*, 2013).

In the telomeric region, the HR mechanism can occur in 3 main pathways: Telomere Sister Chromatid Exchange (T-SCE), T-loop Homologous Recombination (T-*loop* HR), and Alternative Lengthening of Telomeres (ALT). In some insects from the order Diptera that lost the telomerase, a retrotransposon-based mechanism (RM) is used to regenerate chromosomal ends (Mason et al., 2008).

T-SCE is a mechanism for exchanging telomeric sequences between sister chromatids. The mechanism has harmful consequences when unequal crossing-over occurs in the telomeric region, and, in this case, one of the daughter cells inherits a short telomere (Lazzerini-Denchi and Sfeir, 2016). In the T-loop HR, an extrachromosomal duplex or single-stranded circular DNA molecule composed of t-arrays (t-circle) is used in a rolling-circle mechanism (forming σ-form ‘tailed circles’), thus generating long extrachromosomal t-arrays (Tomaska and Nosek, 2009). In the ALT mechanism, the telomeres length maintenance depends on the strands’ recombination without telomerase action (Bryan et al., 1995; Henson et al., 2002; Muntoni and Reddel, 2005). It is believed that in ALT, the single-stranded telomeric termination invades double-stranded telomere sequence or, in the other way, anneals to single-stranded DNA and uses it as a template for the synthesis of a new telomeric DNA sequence (Cesare and Reddel, 2010). The template can come from the own telomere (t-loop formation), sister chromatid or another chromosome telomere, or extrachromosomal telomeric DNA copies (Bryan *et al.*, 1995; Cesare and Reddel, 2010). 

At least, in the RM pathway, a telomeric retrotransposon is transcribed and posteriorly translated in an element-encoded GAG-like protein. GAG binds the retrotransposon RNA, re-entry the nucleus, and attaches to a chromosome end. So, a reverse transcriptase uses the free 3′ hydroxyl group at the chromosome end as a primer to copy the RNA intermediate into the first DNA strand. Second strand synthesis occurs by DNA repair and completes the addition of a new *HeT-A* retrotransposon (for a review, see Mason et al., 2008). 

In some cases, repairing telomere injuries appears harmful to the genome, leading to chromosome fusions and their subsequent breakage during cell proliferation, causing the unequal genetic material heritage among daughter cells. 

## Interstitial telomeric sequences

Interstitial telomeric sequences (ITS) are composed of telomeric motifs located in non-terminal regions of the chromosomes, as in pericentromeric and interstitial regions (between the centromere and telomere) (Meyne et al., 1990; Slijepcevic, 1998; Bolzán and Bianchi, 2006; Ruiz-Herrera et al., 2008). Originally, the ITS occurrence was related to chromosome fusions (Meyne *et al.*, 1990). This kind of ITS located in chromosome fusion points was posteriorly called heterochromatic ITS (het-ITS), which confers chromosome fragility and contributes to genome evolution (Ruiz-Herrera *et al.*, 2008; Bolzán 2012, 2017; Slijepcevic, 2016; Barros et al., 2017; Glugoski et al., 2018). However, different ITS distribution patterns, even in closely related species, reveal their dynamic nature in chromatin composition and epigenetic changes (Swier et al., 2012; Rovatsos et al., 2015). 

According to their organization, location, and flanking sequences, ITS can be classified into four types: short-ITS, subtelomeric-ITS, fusion-ITS, and het-ITS. Short-ITS comprises chromosomal regions generally containing up to 20 TTAGGG tandemly repeated sequences (Azzalin et al., 2001; Nergadze et al., 2004). Based on mammalian genome data, short-ITS are grouped according to their organization and flanking sequences into five subclasses: *(i)* Class A: short-ITS flanked by the same repetitive element on both sides (Short Interspersed Nuclear Elements - SINEs, or Long Interspersed Nuclear Elements - LINEs, for example); *(ii)* Class B: short-ITS is flanked by repetitive units organized in the same direction, in both sides; *(iii)* Class C: short-ITS is flanked by single-copy DNA, in both sides; *(iv)* Class D: short-ITS is flanked by transposable elements in one side and single-copy DNA in another side; *(v)* Class E: short-ITS is located in the junction between two distinct repetitive elements (Azzalin *et al.*, 2001).

Some theories explain short-ITS occurrence by insertion of telomeric repeats during the DSB repairs, being the ITS considered relics of an ancestral break (Nergadze et al., 2004; Ruiz-Herrera et al., 2008). As abovementioned, short-ITS can be added in a canonical NHEJ with telomerase involvement in this pathway (for a review, see Ruiz-Herrera *et al.*, 2008). A second proposal for short-ITS origin involves the possibility of being remnants of a transposable element insertion (Azzalin et al., 2001; Nergadze *et al.*, 2004). Still, the short-ITS may have been simply the birth of a microsatellite containing the telomeric repeat unit, and its expansion or shortening would occur by DNA slippage (Mondello et al., 2000).

The subtelomeric-ITS is composed of thousands of TTAGGG units tandem repeats degenerated into a 5’-3’ organization (head to the tail cluster), which are present in subterminal regions of human chromosomes, as also seen in other vertebrate species (Bolzán, 2017). The proposal for subtelomeric-ITS admits that this degenerate region was originally a true telomere. In this pathway, since a translocation event has established a new telomere, posteriorly, the former telomere sequences degenerate in subtelomeric-ITS (Bolzán, 2017).

The fusion-ITS is a terminology used to describe the TTAGGG repeats flanked by small subtelomeric sequences present at human chromosome 2q13. This fusion-ITS maintain the remnants of end-to-end fusion (or telomere-telomere fusion), related to the evolutionary origin of human chromosome 2 (Azzalin et al., 1997, 2001). Nowadays, fusion-ITS can be figured out in other organisms since genome assembling has expanded over numerous groups.

Het-ITS organize large blocks of telomeric repeats located into the heterochromatin, usually in some chromosomes’ centromeric or pericentromeric regions (Lin and Yan, 2008; Ruiz-Herrera et al., 2008). Telomeric proteins (TRF1, TRF2, and RAP1) could bind in the het-ITS, suggesting that the shelterin complex has an important role in these region’s organization and function (Zakian, 1995; Simonet et al., 2011). Yet, the het-ITS could be relicts of chromosome rearrangements (e.g., Robertsonian - Rb fusion or, pericentric inversion) with fundamental importance in chromosome evolution in some groups (Paço et al., 2013; Matoso Silva et al., 2016; Viana et al., 2016; Deon et al., 2020). 

In the chromosome fusion model, het-ITS are consequences of end-to-end fusion between two dysfunctional telomeres located in ancestral chromosomes (usually in acrocentrics) and posteriorly inactivation of one centromere in the dicentric chromosome formed (Paço et al., 2013; Bolzán, 2017). Nevertheless, in vertebrate chromosomes were visualized that het-ITS are subject to TTAGGG units amplification by unequal crossing-over, DNA slippage, or gene conversion mechanisms, generating large het-ITS blocks in centromeric/pericentromeric chromosome regions (Meyne et al., 1990; Ruiz-Herrera et al., 2008; Schmid and Steinlein, 2016). Once ITS are frequently flanked by satellite DNA and transposable elements, a mechanism based on transposable elements insertion containing telomeric repeats was also proposed in the het-ITS origin (Bolzán, 2017).

Furthermore, subsequent chromosomal rearrangements (inversions, translocations, and fusions) may involve these repeated sequences and redistribute them internally in the chromosomes (Ruiz-Herrera et al., 2008). Finally, chromosomal fissions on ITS, which are naturally prone to breakage, can serve as a substrate for forming a new telomere and generating new acrocentric chromosomes in the genome (Ruiz-Herrera *et al.*, 2008), contributing to karyotype evolution (Bolzán, 2017).

### ITS *in situ* localization method

The usual methodology to detect canonical telomeres and ITS is the fluorescence *in situ* hybridization (FISH) with a telomeric sequence probe (see full method description in supplementary material S1). The general telomeric sequence of the vertebrates is easily amplified by polymerase chain reaction (PCR) using the oligonucleotides (TTAGGG)_5_ and (CCCTAA)_5_, and no template DNA (Ijdo et al., 1991, Figure 5). For insect telomeric motif, the oligonucleotide used is (TTAGG)_5_ and (CCTAA)_5_. In insects, the tyramide signal amplification procedure has been used to detect shorter ITS (Rego and Marec, 2003). In addition, in vertebrates, telomeric sequences detection has been performed using Peptide Nucleic Acid (PNA) probes, or less commonly, by the Primed *in situ* Labeling (PRINS) method (Azzalin et al., 1997, 2001; Ruiz-Herrera et al., 2008; Bolzán, 2017). A rigorous analysis of the chromosomal spreads is crucial in all methods due to the faint signals in some ITS/telomere chromosome markings.

**Figure 5:**
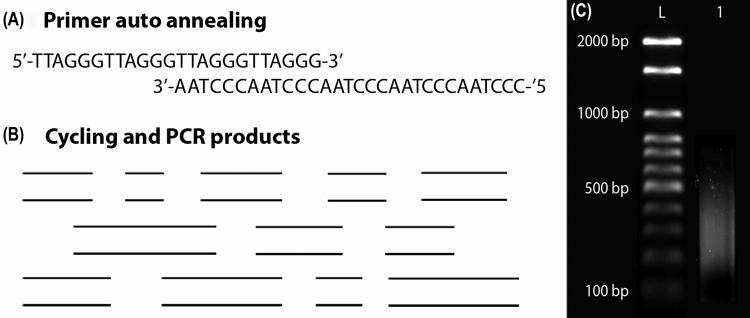
Summary of steps to amplify the general telomeric sequence of the vertebrates (TTAGGG)_n_ by polymerase chain reaction (PCR) using the oligonucleotides (TTAGGG)_5_ and (CCCTAA)_5_, and no template DNA. In (A), a schematic representation of the primer auto annealing, providing a double-strand terminal with free OH; (B) representation of the amplicons with different sizes due to distinct points of telomere units pairing during the PCR; (C) agarose gel 1% showing a smear resulted from the PCR (L = ladder 100 bp; 1 = telomere amplicons with desired sizes, i.e., 100 - 600 bp. For a detailed method, please see Supplementary Material S1.

## ITS and chromosomal remodeling in insects and vertebrates

Terminal telomeric sequences are naturally prone to breakage, leading to chromosome plasticity (Slijepcevic, 2016). In addition, telomere sequences could be considered hotspots for chromosomal breakage when organizing ITS (Slijepcevic *et al.*, 1997). Some studies show that telomeric DNA damage can be irreparable, causing persistent DDR activation (Fumagalli et al., 2012) or remaining as fragile sites (Sfeir et al., 2009). Once ITS could act as an unstable chromosome site, in some animal groups it is noticed chromosome remodeling events as a consequence or cause of the ITS occurrence in their karyotypes. 

### Insects

Insects present extreme variability of karyotypes, chromosome number and morphology, and types of sex chromosome systems (Kaiser and Bachtrog, 2010; Blackmon et al., 2017) due to chromosomal rearrangements, like fusions, fissions, translocations, and inversions. However, only a few ITS cases containing loci of (TTAGG)_n_ were detected (see references below), even in species with highly rearranged karyotypes. They are limited to few species, representatives of orders Lepidoptera (Rego and Marec, 2003), Hemiptera (Chirino et al., 2017), and Orthoptera (López-Fernández et al., 2004; Jetybayev et al., 2012, 2017; Camacho et al., 2015; Grzywacz et al., 2019; Buleu et al., 2020; Warchałowska-Śliwa et al., 2021).

**Figure 6 -:**
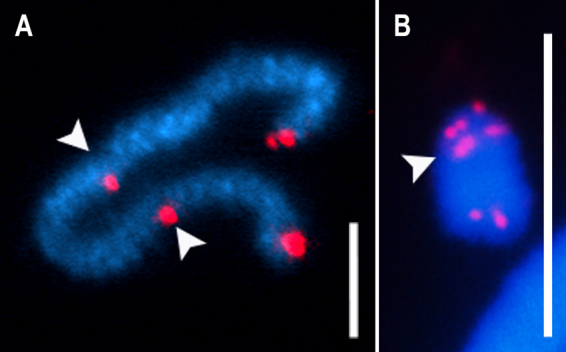
Selected chromosomes showing the occurrence of ITS on insect chromosomes (arrowheads) in addition to regular telomeric signals on the end of chromosomes (in red). In (A), a pachytene bivalent from female stained by TSA-FISH (FISH with Tyramide Signal Amplification, see Carabajal Paladino *et al.*, 2014) from the Lepidoptera *Orgya antiqua* (for details see Marec and Rego, 2003); In (B), *Schistocerca gregaria* (Orthoptera), a small mitotic chromosome from female embryo (for details see Camacho *et al.*, 2015). In all images, the probe used was (TTAGG)_n_. Bar = 5 μm.

In Lepidoptera, ITS were detected only in *Ephestia kuehniella* mutants with fused chromosomes induced from radiation (diploid number - 2n = 59) and in *Orgyia antiqua,* a species with 2n reduction (2n = 28) occasioned by multiple fusions (Figure 6A). Besides typical telomere on chromosome termini in both species, the hybridization signal for (TTAGG)_n_ probe is observed. For *O. antiqua*, the ITSs are probably remnants of multiple chromosomal fusions, but in *E. kuehniella* although the ITS are present in multiple chromosomes, they are not on fused ones (Rego and Marec, 2003).

In hemipterans, ITS motifs were documented in the representatives of giant water bugs *Belostoma* (Belostomatidae). Striking macro-chromosomal variability is observed in this genus as a result of fragmentations and fusions involving autosomes-autosomes and autosomes-sex chromosomes (Chirino and Bressa, 2014; Gallo *et al.*, 2017). Chirino *et al.* (2017), through chromosomal mapping of (TTAGG)_n_ on six species with a different number of autosomes (6, 14, and 26 chromosomes) and simple (XY/XX) and multiple (X_1_X_2_Y/X_1_X_1_X_2_X_2_) sex chromosome systems, revealed the incidence of ITS in the species with 2n reduction. Moreover, these species with reduced 2n presented larger chromosomes. This supported that telomere-telomere fusions were the major chromosomal rearrangement involved in karyotype evolution in *Belostoma* bugs from an ancestral karyotype of 2n = 26+XY/XX.

Among insects, Orthoptera is the group with most ITS cases described until now, with occurrence in more than 20 species. The ITS were noticed in species belonging to multiple groups, including representatives of Tettigoniidae, Pamphagidae, and Acrididae (López-Fernández et al., 2004; Jetybayev et al., 2012, 2017; Camacho et al., 2015; Grzywacz et al., 2019; Buleu et al., 2020; Warchałowska-Śliwa et al., 2021). In this last group, it was present on some representatives from subfamily Gomphocerinae and in *Podisma pedestris* (Cantatopinae), *Eyprepocnemis plorans* (Eyprepocneminae), and *Schistocerca gregaria* (Cyrtacanthacridinae) (Figure 6B). Interestingly, some Acrididae representatives with ITS have 2n = 23, X0 and acrocentric chromosomes, which are ancestral to the group (Husemann et al., 2022), with no apparent macro chromosomal rearrangement. According to Grzywacz *et al.* (2019), in *P. pedestris* (2n = 23), the occurrence of ITS could suggest rearrangements, like inversions, telomere fusion, unequal crossing over, or insertion of telomeric DNA on unstable sites.

A remarkable example of ITS occurrence in autosomes was observed on Gomphocerinae (Acrididae) representatives with the reduced 2n, i.e., 2n = 17. The ITS was reported in *Chorthippus jacobsoni* on the pericentric region of the biarmed pairs (pairs 1-3), revealing that the centric fusion between ancestral chromosomes that originated these pairs was not a Rb rearrangement but a true telomeric fusion that could generate true dicentric chromosomes. On *Aeropus sibiricus*, polymorphic occurrence of ITS was noticed on chromosome six, as a consequence of a paracentric inversion in which the breakpoint involved the true telomeric DNA block. In other species with 2n = 17, no ITS were observed on large metacentric rearranged chromosomes (Jetybayev et al., 2012). In the Tettigoniidae *Gonatoxia helleri,* the occurrence of ITS on all chromosomes seems to be in concordance with points of fusion and inversion rearrangements (Warchałowska-Śliwa et al., 2021).

Besides occurrence on autosomes, the ITS were reported on sex chromosomes of orthopterans as a result of chromosome rearrangements involved in the origin of the neo-XY sex chromosomes. On Pamphagidae, multiple species with neo-XY harbor ITS on the pericentromeric region of neo-X, as a consequence of chromosome fusion between an ancestral autosome and the X chromosome (Jetybayev et al., 2017), although pericentric inversions could also be involved in the posterior origin of the ITS (Buleu et al., 2020). ITS on ancestral X chromosome from X0/XX sex system was scarcely observed (Buleu *et al.*, 2020; Warchałowska-Śliwa et al., 2021). Finally, in multiple species of Orthoptera with neo-sex system, although resultant of fusion chromosome rearrangements no ITS are noticed, suggesting that the Rb fusions involved the loss of telomeres, originated from double chromosome breaks or the ITS were eliminated later along sex chromosomes differentiation. Furthermore, the absence of ITS signals detection could be resulted by the low number copies of telomeric repeats (Palacios-Gimenez et al., 2013, 2015a, 2015b).

### Fishes

In fish species, the ITS were classified into four categories: *(i)* telomeric DNA sequences located at the pericentromeric regions; *(ii)* ITS observed between centromeres and the telomeres located at terminal regions; *(iii)* telomeric DNA sequences that scatter along the nucleolar organizer regions (NORs); and *(iv)* telomeric DNA repeats interspersed with the entire chromosomes (Ocalewicz, 2013). These kinds of ITS were described in species into 12 fish orders (Ocalewicz, 2013). In some groups with chromosomal remodeling, most of the pericentromeric ITS was described as relicts of chromosome fusion events (Rocco et al., 2001, 2002; Chew et al., 2002; Harvey et al., 2002; Milhomem et al., 2008; Ocalewicz *et al.*, 2009; Felippe and Foresti, 2010; Mota-Velasco et al., 2010; Scacchetti et al., 2011; Blanco et al., 2012, 2017; Errero-Porto et al., 2014; Favarato et al., 2016; Barbosa et al., 2017; Barros et al., 2017; Glugoski et al., 2018, 2022; Deon et al., 2022a) or as unstable sites triggering DSBs and chromosome rearrangements (Rosa et al., 2012; Deon *et al.*, 2020, 2022b). In other cases, e.g., in some *Characidium* species, a conserved karyotype with ITS was proposed due to ectopic transposition or events of homologous and non-homologous recombination (Scacchetti *et al.*, 2015; Oliveira et al., 2021a).

ITS considered vestiges of chromosome fusions were also proposed in the origin of the multiple sex chromosome systems in fishes (Cioffi and Bertollo, 2010; Cioffi *et al.*, 2010; Blanco et al., 2017). In *Erythrinus erythrinus* and *Hoplias malabaricus*, the ITS are relicts of chromosome rearrangements on the X_1_X_2_Y sex chromosome system origin (Cioffi and Bertollo, 2010; Cioffi *et al.*, 2010). Chromosome rearrangement with pericentromeric ITS maintaining was also described in the origin of the X chromosome in one *Harttia* lineage possessing XX/XY_1_Y_2_ sex chromosome system (Blanco *et al.*, 2017; Deon et al., 2022a). The data reinforce the proposal of independent origin of multiple sex chromosome systems in some fish groups triggered by chromosome rearrangements without a previous simple sex chromosome system occurrence (Deon *et al.*, 2020; Sassi et al., 2020). 

Acipenseriformes species are characterized by a large number of chromosomes, of which the majority are microchromosomes (Fontana et al., 1998, 2004). In this group, scattered telomeric signals along all microchromosome extensions were reported (Fontana *et al.*, 1998, 2004; Ocalewicz, 2013). Extensive amplification processes extending telomeric arrays to an extraordinary length ranging from 40 kb to 2 Mb, or even longer, were proposed to explain the entire microchromosomes possessing interspaced telomeric DNA sequences (Delany et al., 2000; Ocalewicz, 2013).

**Figure 7 -:**
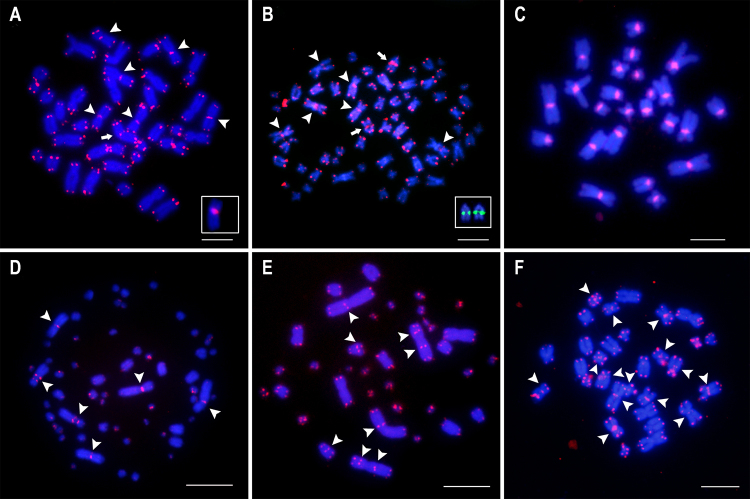
Metaphases of distinct groups of vertebrate species submitted to FISH using (TTAGGG)_n_ probes, evidencing ITS markings (in red). (A) The fish species *Rineloricaria* sp. (Loricariidae) showed a polymorphic karyotype with 2n = 41 chromosomes, the arrowheads showed het-ITS, and the arrow evidenced a chromosome bearing the co-located het-ITS/5S rDNA (box). (B) Another fish species *Harttia* sp. (Loricariidae) (2n = 62) showed 8 het-ITS (arrowheads and arrows), the arrows indicated co-located het-ITS/45S rDNA sites, the chromosome pair bearing 45S rDNA is highlighted in the box. (C) The amphibian *Boana faber* (Hylidae) showed ITS blocks in all chromosomes in the centromeric regions, suggesting that the telomere motifs could compound the centromeric satellite DNA units. (D) The karyotype of the snake *Eunectes murinus* (Boidae) (2n = 36) showed ITSs (arrowheads). (E) The turtle *Phrynops* sp. (Chelidae) (2n = 58) presented ITS distributed in the centromeric and interstitial regions (arrowheads). (F) The bat species *Sturnira lilium* (Phyllostomidae) (2n = 30) showed 14 ITS in the chromosomes. Bar = 10 μm.

ITS are also collocated or adjacent to NORs, usually related to CMA_3_-positive GC-rich heterochromatin in some fish species (Reed and Phillips, 1995; Ocalewicz et al., 2004; Pomianowski et al., 2012; Ocalewicz, 2013; Sember et al., 2015, 2018). Telomeric motifs scattered into NORs were detected in species from Anguilliformes, Mugiliformes, Salmoniformes, Syngnathiformes, and Cypriniformes (Reed and Phillips, 1995; Salvadori et al., 1995; Gornung et al., 2004; Ocalewicz *et al.*, 2004; Rossi et al., 2005; Libertini et al., 2006; Pomianowski *et al.*, 2012; Ocalewicz, 2013; Sember *et al.*, 2015, 2018). The colocalization of ribosomal repeats and telomeric sequences was proposed to stabilize broken chromosomal ends (Pich et al., 1996; Liu and Fredga, 1999). On the other hand, consistent evidence of the association between 5S or 45 rDNA clusters and ITS (collocated or adjacent sites) organizing evolutionary breakpoint regions was proposed in some armored catfish genera (Rosa et al., 2012; Barros et al., 2017; Glugoski et al., 2018; Deon et al., 2020, 2022b). In some species of *Rineloricaria* and *Ancistrus*, the adjacent regions of ITS and 5S rDNA sites organize unstable chromosome sites (Rosa *et al.*, 2012; Barros *et al.*, 2017; Glugoski *et al.*, 2018, 2022; Figure 7A). In addition, *Harttia* species possesses 5S and 45S rDNAs close to or inside ITS promoting extensive chromosomal remodeling in the lineage (Deon *et al.*, 2020; Figure 7B).

### Amphibians

Amphibians are a diverse class of vertebrates, with most cytogenetic studies concentrated in species of the order Anura. Few species of the orders Caudata and Gymnophiona have been investigated cytogenetically, and most of the available data are limited to conventional karyotype descriptions and base-specific fluorochrome staining. To the best of our knowledge, we found only two reports of the chromosomal mapping of the telomeric motif in the karyotype of Caudata species. While the FISH with (TTAGGG)_n_ motif detected the terminal region of all chromosomes of *Bolitoglossa paraenses* (Silva et al., 2014), the chromosomal mapping experiments in *Cynops pyrrhogaster* did not detect any hybridization signal in the karyotype (Murakami et al., 2007). The absence of a hybridization signal with (TTAGGG)_n_ probe in chromosomes of *Cynops pyrrhogaster* suggests the need for future molecular characterization of terminal regions of these chromosomes to understand better these unexpected results (Murakami *et al.*, 2007).

The occurrence of ITS has already been reported in more than 50 species of the order Anura (Schmid and Steinlein, 2016; Schmid *et al.*, 2018; Teixeira et al., 2016; Zattera et al., 2019; Suárez et al., 2020) belonging to the families Centrolenidae, Dicroglossidae, Hylidae, Leptodactylidae, Pelodryadidae, Phyllomedusidae, Pipidae, and Strabomantidae. Despite most of these descriptions, the ITS occurrence could not be explained as a vestige of interchromosomal rearrangement events, but the case observed in *Scarthyla* is one interesting exception. The hypothesis of the 2n reduction from 2n = 24 to 2n = 22 by chromosome fusion event in *Scarthyla* is supported by a conspicuous signal of centromeric ITS in chromosome pair 3 (Suárez *et al.*, 2013). 

The ITS of Anura chromosomes are frequently associated with heterochromatic regions that suggest the (TTAGGG)_n_ sequences are an essential component of repetitive DNA of this group (Schmid and Steinlein, 2016). For example, the karyotype of *Boana faber* showed conspicuous centromeric heterochromatic blocks also FISH-positive with (TTAGGG)_n_ probe in all chromosome pairs (Schmid and Steinlein, 2016; Schmid *et al.*, 2018; Figure 7C). These centromeric segments are AT-rich repetitive sequences and ITS signals, revealing the importance of the telomeric-like motifs to compose these heterochromatic portions (Schmid and Steinlein, 2016). However, large clusters of ITS detected in euchromatic chromosome regions represent a unique feature in some Anuran karyotypes (Schmid and Steinlein, 2016). For example, the karyotype of *Boana boans* showed conspicuous ITS in the non-heterochromatic chromosome region of the short arms of pairs 2 and 9 (Mattos et al., 2014; Schmid and Steinlein, 2016; Schmid *et al.*, 2018). A similar condition was reported to *Phyllomedusa vailantti* (Bruschi et al., 2014) and in the *Sphaenorhynchus lacteus* (Suárez et al., 2013). Also, these chromosomal segments did not reveal heterochromatin features by C-banding or fluorochromes staining (Suárez *et al.*, 2013; Bruschi *et al.*, 2014; Mattos *et al.*, 2014; Schmid *et al.*, 2018).

The screening of karyotypes of natural hybrids of anuran reveals interesting contributions to the distribution and dynamic of ITS in these individuals. For example, *Phyllomedusa distincta* (2n = 26) hybridized with *P. tetraploidea* (2n = 52) in high frequency in one stable secondary contact zone in the Atlantic Forest of Brazil, originating a natural triploid population (3n = 39). Both parental species collected from the hybridization zone showed het-ITS accumulated on centromeric regions of the homologs of pairs 6, 7, and 11, cytogenetic markers present and stable all triploid individuals analyzed (Gruber et al., 2013). Curiously, populations of *P. distincta* collected outside of the hybridization zone showed an identical FISH signal of the homologs of pair 11 but differs by the absence of the het-ITS signal on pairs 6 and 7, and by the additional signal in the centromeric region of pair 8 (Bruschi et al., 2014), reveling an interpopulation variation. Cytogenetic studies of the triploid hybrid (3n = 36) from *Dryophytes chrysosceli* (2n = 24) and *Dryophytes* versicolor (4n = 48) also allow identifying sharing het-ITS from parental in the hybrid karyotype (Wiley et al., 1992). In this case, the polymorphic condition absence/presence of het-ITS in the long arm of chromosome pair 1 reveals interesting founds about the population dynamics of these chromosomal markers. Among individuals of the *D. chrysoscel* from hybridization zone is observed in heterozygous (+/-) and homozygous (+/+ and -/-) to the condition of chromosome pair 1 while in *D. vesicolor*, tetraploid species, exhibits individuals with complete absence of ITS signal (homozygous -/-/-/-) or with only two chromosomes of the 4 homologs with FISH-signal (heterozygous +/+/-/-) (Wiley *et al.*, 1992). The karyotype of the natural triploid hybrid between this species exhibits only one chromosome 1 with ITS signal (Wiley *et al.*, 1992). 

### Reptiles

The karyotype of non-avian reptiles exhibits complex chromosomal evolution scenarios and a recent accumulation of knowledge about ITS distribution. The Archosauromorpha included the turtle sister group of the crocodile+birds. The species of order Crocodylia show karyotype composed exclusively of macrochromosomes probably due to fusions between microchromosomes that resulted in the disappearance of all microchromosomes in this lineage, estimated around 230 Mya (Deakin and Ezaz, 2019). Currently, just six crocodilian species were analyzed by chromosomal mapping with (TTAGGG)_n_ probe: *Crocodylus siamensis* (Kawagoshi et al., 2008), *Caiman latirostris*, *Caiman crocodiles crocodiles*, *Paleosuchus palpebrosus*, *Alligator mississippiensis*, and *Aligator sinensis* (Oliveira et al., 2021b). The unique case of the ITS was reported in the karyotype of *Caiman crocodilus crocodiles* (FN = 60; 24t + 18m/sm) from the Amazon region, Brazil (Oliveira *et al.*, 2021b), with (TTAGGG)_n_ signal on centromeric/pericentromeric part of pairs 14, 15, and 16. This karyotype differs from the specimens of *C. crocodilos* from the United States (FN = 62; 22t + 20m/sm), and the presence of ITS could be represented as the signature of the chromosomal rearrangements that occurred during the chromosomal evolution of *C. crocodilos* (Oliveira *et al.*, 2021b). 

Few turtle karyotypes have been reported with ITS. From 65 species cytogenetically analyzed (Clemente et al., 2020, 2021) with this chromosome marker, only ten showed hybridization signals of telomere-like motifs in interstitial regions of chromosomes (Clemente *et al.*, 2020). Curiously, the non-telomeric repeats in turtles were majoritarian detected in the centromeric region of chromosomes (Cavalcante et al., 2018; Clemente *et al.*, 2020; Figure 7D). The unique exception observed was in the karyotype of *Elseya novaeguineae* (Mazzoleni et al., 2020), in which an interesting heteromorphism male-specific was observed with (TTAGGG)_n_ probe, revealing richness of the telomeric-like motifs in the interstitial position of the chromosome Y. In sea turtles, the ITS were also observed on the microchromosomes (Machado et al., 2020a, 2020b). Despite the prevalence of centromeric ITS in turtle chromosomes, when the karyotypes were analyzed in the phylogenetic context, any occurrence of ITS could be assigned as interchromosomal rearrangements (Clemente *et al.*, 2020). The optimization of chromosomal data on phylogenetic trees helped to understand the putative origins of the ITS in genomes, which discarded the “a priori” hypothesis of the intrachromosomal fusions. 

Squamate reptiles include lizards, snakes, and amphisbaenian species. The karyotype of Squamata showed a high variation of macrochromosomes and microchromosomes numbers, including one lineage that showed karyotypes exclusively composed of macrochromosomes. Cytogenetic data were reported on more than 100 species with evidence of ITS in their karyotypes (Rovatsos et al., 2015; Clemente et al. 2020; Kostmann et al., 2020; Augstenová et al., 2021). The non-telomeric (TTAGGG)_n_ motifs are randomly distributed in the centromeric, pericentromeric, and interstitial chromosomes regions (Rovatsos *et al.*, 2015; Figure 7E). They have revealed higher levels of chromosomal diversity predicted by classical cytogenetic studies in this group (Rovatsos *et al.*, 2015).

### Birds

FISH experiments detecting telomeric sequences in birds usually show just terminal signals (Nanda et al., 2002; Nishida et al., 2008; dos Santos et al., 2015, 2017; Rodrigues et al., 2017; Kretschmer et al., 2018), with the interesting finding that more prominent signals are observed in microchromosomes compared to macrochromosomes (Nanda *et al.*, 2002; dos Santos *et al.*, 2015, 2017). On the other hand, ITS are considered vestiges of chromosomal rearrangements that are particularly frequent in the chicken and primitive Palaeognathae birds (Nanda *et al.*, 2002; Nishida *et al.*, 2008), but also have been seen in other bird groups (Nanda *et al.*, 2002; Derjusheva et al., 2004).

Some studies anchored in phylogenetic analyses showed that many ITS observed in Palaeognathae lineage due to ancestral fusions gradually disappeared along with the divergence of Palaeognathae and Neognathae (Nanda et al., 2002; Kretschmer et al., 2018). In some bird species, where ITSs were expected to be present due to tandem chromosome fusions or centric fusions occurrence, it has been proposed that the telomeric DNA was lost during the chromosomal rearrangements (Nanda *et al.*, 2007; de Oliveira *et al.*, 2008; Nishida et al., 2013).

### Mammals

The organization causes and consequences of the ITS occurrence in the human genome are reasonably well understood, as abovementioned. Mammals generally show a vast quantity of studies discussing ITS causes and consequences in chromosome evolution. A concise description of ITS cases in mammal genomes is shown here. In several mammalian groups, the presence of ITS located in the centromere, pericentromere, or those found between the centromere and the telomere were classified into short-ITS, subtelomeric-ITS and het-ITS (Ijdo et al., 1991; Farré et al., 2009; Ventura et al., 2012; Dumas et al., 2016; Matzenbacher et al., 2022). ITS occurrence or het-ITS as a vestige of the chromosomal rearrangement is an usual condition in the main descriptions of mammalian species (Lee et al., 1993; Scherthan, 1995; Metcalfe et al., 1998, 2002, 2004; Zou et al., 2002; Hartmann and Scherthan, 2004; Ventura *et al.*, 2006; Rovatsos et al., 2011; Nagamachi et al., 2013; Colomina et al., 2017; Mazzoleni et al., 2017; Matzenbacher *et al.*, 2022, among others). Despite the het-ITS indicating a chromosome rearrangement, some studies also demonstrate the occurrence of telomeric repeats constituting a new component of the satellite DNA in the genomes (Faravelli et al., 2002; Rovatsos *et al.*, 2011).

The Indian muntjac deer karyotype is differentiated by tandem fusion, a rare evolutionary chromosome rearrangement, leading to an extremely reduced karyotype of 6/7 (female/male) chromosomes (Lee et al., 1993; Frönicke and Scherthan, 1997). Posteriorly, Hartmann and Scherthan (2004) proposed that telomere and GC-rich satellite DNA sequences were involved during muntjac chromosome fusions. In addition to deer, ITS and chromosome changes were proposed in other mammalian groups: Chiroptera (Calixto et al., 2014), Perissodactyla (Danielak-Czech et al., 2013), marsupials (Metcalfe et al., 1998, 2002, 2004), primates (Dumas et al., 2016; Mazzoleni et al., 2017), and Rodentia (Ventura et al., 2006; Rovatsos et al., 2011; Nagamachi et al., 2013; Lanzone et al., 2015; Colomina et al., 2017).

In Phyllostomidae bats, the het-ITS were proposed as vestiges of Rb fusion during the chromosomal evolution (Calixto et al., 2014). Unstable chromosome regions with t(7;13)(q13;q46) reciprocal translocation showed an ITS as a relict of the chromosomal rearrangement in pigs (Danielak-Czech et al., 2013). In Australian marsupial, the distribution of the (TTAGGG)_n_ sequence into moderate and large centromeric heterochromatin blocks reflect its presence as a native component of satellite DNA rather than its involvement in past rearrangements (Metcalfe et al., 2004). On the other hand, in marsupial karyotypes with little heterochromatin, the ITS was proposed as relicts of chromosome rearrangements and 2n reduction (Metcalfe *et al.*, 2007).

Subtelomeric-ITS and het-ITS have been proposed in some primate genomes (Meyne et al., 1990; Garagna et al., 1997; Go et al., 2000; Azzalin et al., 2001; Hirai, 2001; Ruiz-Herrera et al., 2002, 2005; Wijayanto et al., 2005; Mudry et al., 2007; Dumas et al., 2016; Mazzoleni et al., 2017). Among the Old World monkeys (Cercopithecini), a centromeric het-ITS in *C. petaurista* and *C. guereza* (Colobini) was described (Mazzoleni *et al.*, 2017). ITS were not observed in *Hylobates lar* and *Pongo pygmaeus*, while *Macaca fascicularis* (Papionini), *Pan paniscus*, and *Pan troglodytes* (Hominoidea) have multiple het-ITS (Azzalin *et al.*, 2001; Hirai, 2001; Hirai *et al.*, 2005; Ruiz-Herrera *et al.*, 2005; Mazzoleni *et al.*, 2017). Pericentromeric het-ITS and many large telomeric/subtelomeric signals, presumably resultant of the amplification of telomeric sequences were described in *Lemur catta* (Mazzoleni *et al.*, 2017). In other Lemuriformes, many interspersed telomeric sites (het-ITS) were observed in the karyotypes (Meyne *et al.*, 1990; Garagna *et al.*, 1997; Go *et al.*, 2000; Mazzoleni *et al.*, 2017).

Neotropical monkeys (Platyrrhini) characterized by highly derived karyotypes show no or few het-ITS, while species with less reshuffled karyotypes in terms of interchromosomal rearrangements present many het-ITS (Ruiz-Herrera et al., 2005; Mudry et al., 2007; Dumas et al., 2016; Mazzoleni et al., 2017). Some New World monkey species also have ITS in their karyotypes (Ruiz-Herrera *et al.*, 2002, 2005; Dumas *et al.*, 2016), sometimes without heterochromatin correspondence (Mazzoleni *et al.*, 2017). Based on the ITS *in situ* localization in several primate groups, Mazzoleni *et al.* (2017) suggested a correlation between ITS and rearrangements in many species, thus correlating with chromosomal plasticity.

An extensive (TTAGGG)_n_ *in situ* localization demonstrated that pericentromeric het-ITS are a common feature in arvicolid rodents allied to examples of het-ITS amplification at non-pericentromeric regions, and some descriptions of short-ITS at the euchromatic regions (Rovatsos et al., 2011). In the same study, Rovatsos *et al.* (2011) proposed no direct correlation between the presence or absence of het-ITS and the genus or subgenus classification of the Arvicolinae, in which the variation and amplification of ITS occurred independently in each species. Yet, het-ITS have played a significant role in karyotypic variation and evolution of Arvicolinae species, but het-ITS cannot explain the rearrangements that occurred during the karyotype evolution of *Chionomys*, *Arvicola*, and *Microtus* (Rovatsos *et al.*, 2011). In *Cerradomys* (Sigmodontinae), ITS accumulate at the breakpoints, although the possibility of resulting from old fusions was not ruled out (Nagamachi et al., 2013).

Het-ITS repeats and a satellite DNA (named CH5) located in centromeric heterochromatin were described in the chinese hamster (Faravelli et al., 2002). In other rodents, het-ITS have been localized within or at the margins of constitutive heterochromatin (Meyne et al., 1990; Vermeesch et al., 1996; Ono and Yoshida, 1997; Go et al., 2000). In arvicolids, a co-distribution for het-ITS and Msat-160 satellites has been proposed in centromeric heterochromatin’s organization and structure (Rovatsos et al., 2011). 

In the African pygmy mice, *Mus* species, a large amplification of telomeric repeats was identified in the pericentromeric region of acrocentric and most metacentric chromosomes (Colomina et al., 2017). According to the authors, *Mus musculus domesticus* has a different Rb fusion mechanism than African pygmy mice. The number of telomere repeats in the ITS could be a signature of the Rb fusion age of formation (Colomina *et al.*, 2017). Yet, the large amplification of TTAGGG repeats in pericentromeric regions of the acrocentric chromosomes in African pygmy mice were proposed to mediate the formation of Rb fusions (Colomina *et al.*, 2017). At least, the occurrence of the ITS in the differentiation of the sex chromosomes was described in Arvicolinae species (Rovatsos et al., 2011) and in the sex-autosome fusion in African pygmy from the *Mus* genus (Colomina *et al.*, 2017). All data presented here demonstrated the extensive ITS participation in mammal chromosome remodeling events.

## Genomic instability, ITS, and chromosomal rearrangements

Although ITS do not organize functional telomeres and their functions are not entirely elucidated, many studies indicate that ITS plays a fundamental role in the genomic instability and chromosomal evolution in several groups (Perry et al., 2004; Ruiz-Herrera et al., 2008; Slijepcevic, 2016; Bolzán, 2017). Besides ITS presence in species possessing a highly rearranged karyotype, ITS also occurs in close relationship species showing a conservative chromosome structure, i.e., ancestral karyotypes (Nergadze et al., 2004; Swier et al., 2012). In general, the ITS are hotspots for chromosome breaks, recombination, chromosomal rearrangements, amplification sites, and thus, organizing genomic instability sites (Perry *et al.*, 2004; Bolzán, 2017). The nucleotide sequence feature of the ITS contributes to genomic instability (Perry *et al.*, 2004). The guanine-rich segment could organize DNA secondary structures prone to break, triggering chromosomal rearrangements (Salvati et al., 2010; Vannier et al., 2012). 

It is known that het-ITS organize unstable genomic sites, while this role remains controversial in short-ITS (Ruiz-Herrera et al., 2008). Short-ITS are unable to bind telomere proteins or organize complex structures, a condition to prone DSB sites (Ruiz-Herrera *et al.*, 2008). Nevertheless, a study proposed that even short-ITS possessing TTAGGG repetition in minus 100 bp interval are related to genomic recombination increase (Kong et al., 2010, 2013). Dysfunctional telomeres also are considered unstable genomic sites since the inactivation or telomere loss are characteristics for triggering Rb fusions (Slijepcevic, 1998, 2016; Bolzán, 2017). 

Rb fusions are consequences of telomere shortening, centromere chromosome breaks, or telomere inactivation (Meyne et al., 1990; Slijepcevic, 2016; Bolzán, 2017). Still, there are three causes of loss of telomeric function without complete loss of telomere sequences: *(i)* telomeric proteins inactivation; *(ii)* loss of telomere function; and *(iii)* loss of telomerase function (Slijepcevic, 1998). Bolzán (2017) described chromatin conformation’s central role in ITS stability. The nucleosome organization in the telomeric chromatin is around 40 bp shorter than nuclear nucleosomes (Tommerup et al., 1994; Lejnine et al., 1995), and the high compacted regions in het-ITS results in DNA bents, unpaired segments, and DSBs (Rivero et al., 2004). 

Chromatin changes related to epigenetic modifications significantly influence telomere and ITS stability (Gonzalo et al., 2006; Lin and Yan, 2008; Slijepcevic, 2016; Bolzán, 2017). Hypermethylation states help ITS stability, while demethylated or hypomethylated ITS tend to be unstable (Lin and Yan, 2008) favoring telomeric sequences recombination (Gonzalo *et al.*, 2006). Still, the correct association of shelterin complex in ITS helps with chromatin stability, decreasing the unequal crossing-over events between telomeric sequences (Zakian, 1995; Mignon-Ravix et al., 2002; Yang et al., 2011; Bolzán, 2017) and, on the other hand, lacking one or more shelterin associations, the ITS instability increases (Slijepcevic, 2016; Lin and Yan, 2008).

Telomeres ensure the correct anchoring of chromosomes on the nuclear membrane internal surface, usually interacting with the nuclear lamin A/C protein, but ITS association with the nuclear matrix is unclear (Wood et al., 2014). On the other hand, ITS association with end chromosome telomere sequences described as Interstitial Telomeric Loops (ITLs) depend on the TRF2 and nuclear lamin A/C protein binding (Wood *et al.*, 2015). The ITLs act in the telomere stability, gene expression regulation of closely located genes and ITL, and the interaction mechanism with the nuclear membrane (Robin et al., 2014, 2015; Wood *et al.*, 2014, 2015; Robin and Magdinier, 2016).

**Figure 8 -:**
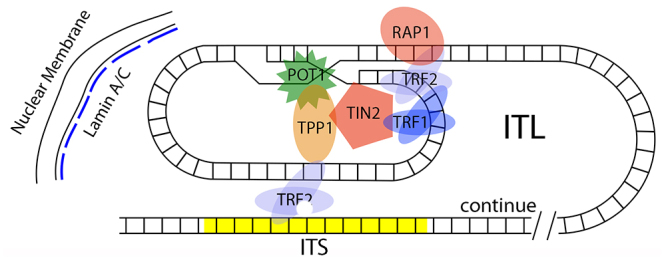
Scheme representing the interstitial telomeric loop (ITL) interacting with interstitial telomere sequences (ITS). TRF2 could facilitate the association between ITL and ITS (yellow). This kind of interaction could generate DSBs, thus triggering chromosome rearrangements.

The ITS/telomere interaction could result in chromosome rearrangements (Figure 8). This interaction was also proposed to cause terminal inversions, reshuffling the gene locations on the chromosomes, thus, promoting gene expression modifications (Robin et al., 2014, 2015; Wood et al., 2014, 2015; Robin and Magdinier, 2016; Bolzán, 2017). The ITLs organization far away from the chromosome ends also acts on the chromosome condensation during mitosis (Wood *et al.*, 2014, 2015). Through all the features presented of the ITS/telomere association, it is evident that these structures are important for chromosomal remodeling (Wood *et al.*, 2014, 2015; Bolzán, 2017).

## Perspectives

Characterizing the telomere motifs and structure is still necessary, mainly for several non-vertebrate groups. Advances in genome assembling in non-model organisms could figure out distinct telomeric motifs. In addition, the studies about chromosomal remodeling involving telomere sequences are incipient in lower vertebrates, besides a vast group of insects and other invertebrates. The participation of het-ITS in chromosome changes in these groups is emerging, but the data still need more robust information on DNA sequencing and epigenetic modifications. ITS trigger DSBs, transpositions, inversions, translocations, and Rb rearrangements in species groups with highly rearranged karyotypes, making the ITS and chromosome ends a central subject of the genomic instability. Thus, advances in ITS characterization are necessary. For that, the DNA sequence characterization over ITS segments, the recognition of telomeric proteins binding and loops formation between telomere and ITS, the evaluation of ITS types, and their epigenetic modifications, allied to *in situ* characterization, could illuminate the karyotype evolution in many groups.

## Supplementary material

The following online material is available for this article:

Supplementary Material S1 - Telomere probe amplification by PCR.

## References

[B1] Aksenova AY, Mirkin SM (2019). At the beginning of the end and in the middle of the beginning: structure and maintenance of telomeric DNA repeats and interstitial telomeric sequences. Genes.

[B2] Anand RP, Shah KA, Niu H, Sung P, Mirkin SM, Freudenreich CH (2012). Overcoming natural replication barriers: Differential helicase requirements. Nucleic Acids Res.

[B3] Anand RP, Lovett ST, Haber JE (2013). Break-induced DNA replication. Cold Spring Harb Perspect Biol.

[B4] Augstenová B, Pensabene E, Veselý M, Kratochvíl L, Rovatsos M (2021). Are geckos special in sex determination? Independently evolved differentiated ZZ/ZW sex chromosomes in Carphodactylid Geckos. Genome Biol Evol.

[B5] Azzalin CM, Mucciolo E, Bertoni L, Giulotto E (1997). Fluorescence in situ hybridization with a synthetic (T2AG3)n polynucleotide detects several intrachromosomal telomere-like repeats on human chromosomes. Cytogenet Cell Genet.

[B6] Azzalin CM, Nergadze SG, Giulotto E (2001). Human intrachromosomal telomeric-like repeats: sequence organization and mechanisms of origin. Chromosoma.

[B7] Azzalin CM, Reichenbach P, Khoriauli L, Giulotto E, Lingner J (2007). Telomeric repeat containing RNA and RNA surveillance factors at mammalian chromosome ends. Science.

[B8] Bah A, Gilson E, Wellinger RJ (2011). Telomerase is required to protect chromosomes with vertebrate-type T2AG3 3’ ends in Saccharomyces cerevisiae. J Biol Chem.

[B9] Balk B, Maicher A, Dees M, Klermund J, Luke-Glaser S, Bender K, Luke B (2013). Telomeric RNA-DNA hybrids affect telomere-length dynamics and senescence. Nat Struct Mol Biol.

[B10] Barbosa P, Pucci MB, Nogaroto V, Almeida MC, Artoni RF, Vicari MR (2017). Karyotype analysis of three species of Corydoras (Siluriformes: Callichthyidae) from southern Brazil: rearranged karyotypes and cytotaxonomy. Neotrop Ichthyol.

[B11] Barros AV, Wolski MAV, Nogaroto V, Almeida MC, Moreira-Filho O, Vicari MR (2017). Fragile sites, dysfunctional telomere and chromosome fusions: What is 5S rDNA role?. Gene.

[B12] Bhargava R, Onyango DO, Stark JM (2016). Regulation of single-strand annealing and its role in genome maintenance. Trends Genet.

[B13] Blackburn EH, Greider CW, Szostak JW (2006). Telomeres and telomerase: the path from maize, Tetrahymena and yeast to human cancer and aging. Nature Med.

[B14] Blackmon H, Ross L, Bachtrog D (2017). Sex determination, sex chromosomes, and karyotype evolution in insects. J Hered.

[B15] Blanco DR, Vicari MR, Artoni RF, Traldi JB, Moreira-Filho O (2012). Chromosomal characterization of armored catfish Harttia longipinna (Siluriformes, Loricariidae): First report of B chromosomes in the genus. Zool Sci.

[B16] Blanco DR, Vicari MR, Lui RL, Traldi JB, Bueno V, Martinez JF, Brandão H, Oyakawa OT, Moreira-Filho O (2017). Karyotype diversity and evolutionary trends in armored catfish species of the genus Harttia (Siluriformes: Loricariidae). Zebrafish.

[B17] Bolzán AD (2012). Chromosomal aberrations involving telomeres and interstitial telomeric sequences. Mutagenesis.

[B18] Bolzán AD (2017). Interstitial telomeric sequences in vertebrate chromosomes: origin, function, instability and evolution. Mutat Res Rev Mutat Res.

[B19] Bolzán AD, Bianchi MS (2006). Telomeres interstitial telomeric repeat sequences, and chromosomal aberrations. Mutat Res.

[B20] Bolzán AD, Páez GL, Bianchi MS, Bianchi NO (2000). Analysis of telomeric repeats and telomerase activity in human colon carcinoma cells with gene amplification. Cancer Genet Cytogenet.

[B21] Bruschi DP, Rivera M, Lima AP, Zuñiga AB, Recco-Pimentel SM (2014). Interstitial telomeric sequences (ITS) and major rDNA mapping reveal insights into the karyotypical evolution of neotropical leaf frogs species (Phyllomedusa, Hylidae, Anura). Mol Cytogenet.

[B22] Bryan TM, Englezou A, Gupta J, Bacchetti S, Reddel RR (1995). Telomere elongation in immortal human cells without detectable telomerase activity. EMBO J.

[B23] Buleu O, Jetybayev I, Mofidi-Neyestanak M, Bugrov A (2020). Karyotypes diversity in some Iranian Pamphagidae grasshoppers (Orthoptera, Acridoidea, Pamphagidae): New insights on the evolution of the neo-XY sex chromosomes. Comp Cytogenet.

[B24] Calixto MS, Andrade IS, Cabral-de-Mello DC, Santos N, Martins C, Loreto V, Souza MJ (2014). Patterns of rDNA and telomeric sequences diversification: contribution to repetitive DNA organization in Phyllostomidae bats. Genetica.

[B25] Camacho JPM, Ruiz-Ruano FJ, Martín-Blázquez R, López-León MD, Cabrero J, Lorite P, Cabral-de-Mello DC, Bakkali M (2015). A step to the gigantic genome of the desert locust: chromosome sizes and repeated DNAs. Chromosoma.

[B26] Carabajal Paladino LZ, Nguyen P, Šíchová J, Marec F (2014). Mapping of single-copy genes by TSA-FISH in thecodling moth,Cydia pomonella. BMC Genet.

[B27] Cavalcante MG, Bastos CEMC, Nagamachi CY, Pieczarka JC, Vicari MR, Noronha RCR (2018). Physical mapping of repetitive DNA suggests 2n reduction in Amazon turtles Podocnemis (Testudines: Podocnemididae). PloS One.

[B28] Ceccaldi R, Rondinelli B, D’Andrea AD (2016). Repair pathway choices and consequences at the double-strand break. Trends Cell Biol.

[B29] Cesare AJ, Reddel RR (2010). Alternative lengthening of telomeres: models, mechanisms and implications. Nat Rev Genet.

[B30] Chawla R, Azzalin CM (2008). The telomeric transcriptome and SMG proteins at the crossroads. Cytogenet Genome Res.

[B31] Chen LY, Lingner J (2013). CST for the grand finale of telomere replication. Nucleus.

[B32] Chew JSK, Oliveira C, Wright JM, Dobson MJ (2002). Molecular and cytogenetic analysis of the telomeric (TTAGGG)n repetitive sequences in the Nile Tilapia, Oreochromis niloticus (Teleostei: Cichlidae). Chromosoma.

[B33] Chirino MG, Bressa MJ (2014). Karyotype evolution in progress: A new diploid number in Belostoma candidulum (Heteroptera: Belostomatidae) from Argentina leading to new insights into its ecology and evolution. Eur J Entomol.

[B34] Chirino MG, Dalíková M, Marec F, Bressa MJ (2017). Chromosomal distribution of interstitial telomeric sequences as signs of evolution through chromosome fusion in six species of the giant water bugs (Hemiptera, Belostoma). Ecol Evol.

[B35] Cioffi MB, Bertollo LAC (2010). Initial steps in XY chromosome differentiation in Hoplias malabaricus and the origin of an X1X2Y sex chromosome system in this fish group. Heredity (Edinb).

[B36] Cioffi MB, Martins C, Bertollo LAC (2010). Chromosome spreading of associated transposable elements and ribosomal DNA in the fish Erythrinus erythrinus. Implications for genome change and karyoevolution in fish. BMC Evol Biol.

[B37] Clemente L, Mazzoleni S, Bellavia EP, Augstenová B, Auer M, Praschag P, Protiva T, Velenský PWP, Fritz U, Kratochvíl L (2020). Interstitial telomeric repeats are rare in turtles. Genes.

[B38] Clemente L, Mazzoleni S, Pensabene E, Protiva T, Wagner P, Fritz U, Kratochvíl L, Rovatsos M (2021). Cytogenetic analysis of the Asian box turtles of the genus Cuora (Testudines, Geoemydidae). Genes.

[B39] Colomina V, Catalan J, Britton-Davidian J, Veyrunes F (2017). Extensive amplification of telomeric repeats in the karyotypically highly diverse African pygmy mice. Cytogenet Genome Res.

[B40] Court R, Chapman L, Fairall L, Rhodes D (2005). How the human telomeric proteins TRF1 and TRF2 recognize telomeric DNA: a view from high-resolution crystal structures. EMBO Rep.

[B41] Croteau DL, Popuri V, Opresko PL, Bohr VA (2014). Human RecQ helicases in DNA repair, recombination, and replication. Annu Rev Biochem.

[B42] Danielak-Czech B, Rejduch B, Kozubska-Sobocińska A (2013). Identification of telomeric sequences in pigs with rearranged karyotype using PRINS technique. Ann Anim Sci.

[B43] de Lange T (2002). Protection of mammalian telomeres. Oncogene.

[B44] de Lange T (2005). Shelterin: the protein complex that shapes and safeguards human telomeres. Genes Dev.

[B45] De Oliveira EH, de Moura SP, dos Anjos LJ, Nagamachi CY, Pieczarka JC, O’Brien PCM, Ferguson-Smith MA (2008). Comparative chromosome painting between chicken and spectacled owl (Pulsatrix perspicillata): Implications for chromosomal evolution in the Strigidae (Aves, Strigiformes). Cytogenet Genome Res.

[B46] Deakin JE, Ezaz T (2019). Understanding the evolution of reptile chromosomes through applications of combined cytogenetics and genomics approaches. Cytogenet Genome Res.

[B47] Delany ME, Krupkin AB, Miller MM (2000). Organization of telomere sequences in birds: evidence for arrays of extreme length and for in vivo shortening. Cytogenet Cell Genet.

[B48] Deng Z, Norseen J, Wiedmer A, Riethman H, Lieberman PM (2009). TERRA RNA binding to TRF2 facilitates heterochromatin formation and ORC recruitment at telomeres. Mol Cell.

[B49] Deon GA, Glugoski L, Vicari MR, Nogaroto V, Sassi FMC, Cioffi MB, Liehr T, Bertollo LAC, Moreira-Filho O (2020). Highly rearranged karyotypes and multiple sex chromosome systems in armored catfishes from the Genus Harttia (Teleostei, Siluriformes). Genes.

[B50] Deon GA, Glugoski L, Hatanaka T, Sassi FDMC, Nogaroto V, Bertollo LAC, Liehr T, Al-Rikabi A, Moreira-Filho O, Cioffi MB, Vicari MR (2022). Evolutionary breakpoint regions and chromosomal remodeling in Harttia (Siluriformes: Loricariidae) species diversification. Genet Mol Biol.

[B51] Deon GA, Glugoski L, Sassi FDMC, Hatanaka T, Nogaroto V, Bertollo LAC, Liehr T, Al-Rikabi A, Moreira-Filho O, Cioffi MB, Vicari MR (2022). Chromosomal rearrangements and origin of the multiple XX/XY1Y2 sex chromosome system in Harttia species (Siluriformes: Loricariidae). Front Genet.

[B52] Derjusheva S, Kurganova A, Haberman F, Gaginskaia E (2004). High chromosome conservation detected by comparative chromosome painting in chicken, pigeon and passerine birds. Chromosome Res.

[B53] Diede SJ, Gottschling DE (1999). Telomerase-mediated telomere addition in vivo requires DNA primase and DNA polymerases alpha and delta. Cell.

[B54] dos Santos MS, Kretschmer R, Silva FA, Ledesma MA, O’Brien PCM, Ferguson-Smith MA, Garnero ADV, de Oliveira EHC, Gunski RJ (2015). Intrachromosomal rearrangements in two representatives of the genus Saltator (Thraupidae, Passeriformes) and the occurrence of heteromorphic Z chromosomes. Genetica.

[B55] dos Santos MS, Kretschmer R, Frankl-Vilches C, Bakker A, Gahr M, O’Brien PCM, Ferguson-Smith MA, de Oliveira EHC (2017). Comparative cytogenetics between two important songbird, models: The zebra finch and the canary. PloS One.

[B56] Dumas F, Cuttaia H, Sineo L (2016). Chromosomal distribution of interstitial telomeric sequences in nine neotropical primates (Platyrrhini): Possible implications in evolution and phylogeny. J Zool Syst Evol Res.

[B57] Errero-Porto F, Vieira MMR, Barbosa LM, Borin-Carvalho LA, Vicari MR, Portela-Castro ALB, Martins-Santos IC (2014). Chromosomal polymorphism in Rineloricaria lanceolata Gunther, 1868 (Loricariidae: Loricariinae) of the Paraguay basin (Mato Grosso do Sul, Brazil): evidence of fusions and their consequences in the population. Zebrafish.

[B58] Faravelli M, Azzalin CM, Bertoni L, Chernova O, Attolini C, Mondello C, Giulotto E (2002). Molecular organization of internal telomeric sequences in Chinese hamster chromosomes. Gene.

[B59] Farré M, Ponsà M, Bosch M (2009). Interstitial telomeric sequences (ITSs) are not located at the exact evolutionary breakpoints in primates. Cytogenet Genome Res.

[B60] Favarato RM, Da Silva M, Oliveira RR, Artoni RF, Feldberg E, Matoso DA (2016). Cytogenetic diversity and the evolutionary dynamics of rDNA genes and telomeric sequences in the Ancistrus genus (Loricariidae: Ancistrini). Zebrafish.

[B61] Felippe CL, Foresti ATL (2010). Evidence of chromosome fusion in Gymnotus sylvius Albert & Fernandes-Matoli, 1999 (Teleostei: Gymnotiformes) detected by telomeric probes and R-banding. Caryologia.

[B62] Fontana F, Lanfredi M, Chicca M, Aiello V, Rossi R (1998). Localization of repetitive telomeric sequences (TTAGGG)n in four sturgeon species. Chromosome Res.

[B63] Fontana F, Bruch RM, Binkowski FP, Lanfredi M, Chicca M, Beltrami N, Congiu L (2004). Karyotype characterization of the lake sturgeon, Acipenser fulvescens (Rafinesque 1817) by chromosome banding and fluorescent in situ hybridization. Genome.

[B64] Fouché N, Cesare AJ, Willcox S, Ozgür S, Compton AS, Griffith JD (2006). The basic domain of TRF2 directs binding to DNA junctions irrespective of the presence of TTAGGG repeats. J Biol Chem.

[B65] Frönicke L, Scherthan H (1997). Zoo-fluorescence in situ hybridization analysis of human and Indian muntjac karyotypes (Muntiacus muntjac vaginalis) reveals satellite DNA clusters at the margins of conserved syntenic segments. Chromosome Res.

[B66] Frydrychová R, Grossmann P, Trubač P, Vítková M, Marec F (2004). Phylogenetic distribution of TTAGG telomeric repeats in insects. Genome.

[B67] Fujiwara H, Osanai V, Matsumoto T, Kojima K (2005). Telomere-specific non-LTR retrotransposons and telomere maintenance in the silkworm, Bombyx mori. Chromosome Res.

[B68] Fumagalli M, Rossiello F, Clerici M, Barozzi S, Cittaro D, Kaplunov JM, Bucci G, Dobreva M, Matti V, Beausejour CM (2012). Telomeric DNA damage is irreparable and causes persistent DNA-damage-response activation. Nat Cell Biol.

[B69] Galati A, Micheli E, Cacchione S (2013). Chromatin structure in telomere dynamics. Front Oncol.

[B70] Gallo RB Aguiar RCM, Ricietto APS Vilas-Boas L, da Silva CRM Ribeiro, JRI Da Rosa R (2017). A new approach to chromosomal evolution in the giant water bug (Heteroptera: Belostomatidae). J Hered.

[B71] Gao H, Cervantes RB, Mandell EK, Otero JH, Lundblad V (2007). RPA-like proteins mediate yeast telomere function. Nat Struct Mol Biol.

[B72] Garagna S, Ronchetti E, Mascheretti S, Crovella S, Formenti D, Rumpler Y, Manfredi Romanini MG (1997). Non-telomeric chromosome localization of (TTAGGG)n repeats in the genus Eulemur. Chromosome Res.

[B73] Garavís M, González C, Villasante A (2013). On the origin of the eukaryotic chromosome: The role of noncanonical DNA structures in telomere evolution. Genome Biol Evol.

[B74] Geronimo CL, Zakian VA (2016). Getting it done at the ends: Pif1 family DNA helicases and telomeres. DNA Repair.

[B75] Glugoski L, Giuliano-Caetano L, Moreira-Filho O, Vicari MR, Nogaroto V (2018). Co-located hAT transposable element and 5S rDNA in an interstitial telomeric sequence suggest the formation of Robertsonian fusion in armored catfish. Gene.

[B76] Glugoski L, Nogaroto V, Deon GA, Azambuja M, Moreira-Filho O, Vicari MR (2022). Enriched tandemly repeats in chromosomal fusion points of Rineloricaria latirostris (Boulenger, 1900) (Siluriformes: Loricariidae). Genome.

[B77] Go Y, Rakotoariso G, Kawamoto Y, Randrianjafy A, Koyama N, Hirai H (2000). PRINS analysis of the telomeric sequence in seven lemurs. Chromosome Res.

[B78] Gómez-González B, García-Rubio M, Bermejo R, Gaillard H, Shirahige K, Marín A, Foiani M, Aguilera A (2011). Genome-wide function of THO/TREX in active genes prevents R-loop-dependent replication obstacles. EMBO J.

[B79] Gonzalo S, Jaco I, Fraga MF, Chen T, Li E, Esteller M, Blasco A (2006). DNA methyltransferases control telomere length and telomere recombination in mammalian cells. Nat Cell Biol.

[B80] Gornung E, Mannarelli ME, Rossi AR, Sola L (2004). Chromosomal evolution in Mugilidae (Pisces, Mugiliformes): FISH mapping of the (TTAGGG)n telomeric repeat in the six Mediterranean mullets. Hereditas.

[B81] Greider CW, Blackburn EH, Greider CW (1995). Telomeres.

[B82] Griffith J, Comeau L, Rosenfield S, Stansel R, Bianchi A, Moss H, de Lange T (1999). Mammalian telomeres end in a large duplex loop. Cell.

[B83] Gruber SL, Silva APZ, Haddad CFB, Kasahara S (2013). Cytogenetic analysis of Phyllomedusa distincta Lutz, 1950 (2n = 2x = 26), P. tetraploidea Pombal and Haddad, 1992 (2n = 4x = 52), and their natural triploid hybrids (2n = 3x = 39) (Anura, Hylidae, Phyllomedusinae). BMC Genet.

[B84] Grzywacz B, Tatsuta H, Bugrov AG, Warchałowska-Śliwa E (2019). Cytogenetic markers reveal a reinforcement of variation in the tension zone between chromosome races in the brachypterous grasshopper Podisma sapporensis Shir. on Hokkaido Island. Sci Rep.

[B85] Hartmann N, Scherthan H (2004). Characterization of ancestral chromosome fusion points in the Indian muntjac deer. Chromosoma.

[B86] Harvey SC, Campos-Ramos R, Kennedy DD, Ezaz MT, Bromage NR, Griffin DK, Penman DJ (2002). Karyotype evolution in Tilapia: mitotic and meiotic chromosome analysis of Oreochromis karongae and O. niloticus × O. karongae hybrids. Genetica.

[B87] Henson JD, Neumann AA, Yeager TR, Reddel RR (2002). Alternative lengthening of telomeres in mammalian cells. Oncogene.

[B88] Heyer WD (2015). Regulation of recombination and genomic maintenance. Cold Spring Harb Perspect Biol.

[B89] Hines WC, Fajardo AM, Joste NE, Bisoffi M, Griffith JK (2005). Quantitative and spatial measurements of telomerase reverse transcriptase expression within normal and malignant human breast tissues. Mol Cancer Res.

[B90] Hirai H (2001). Relationship of telomere sequence and constitutive heterochromatin in the human and apes as detected by PRINS. Methods Cell Sci.

[B91] Hirai H, Matsubayashi K, Kumazaki K, Kato A, Maeda N, Kim HS (2005). Chimpanzee chromosomes: retrotransposable compound repeat DNA organization (RCRO) and its influence on meiotic prophase and crossing-over. Cytogenet Genome Res.

[B92] Husemann M, Dey LS, Sadílek D, Ueshima N, Hawlitschek O, Song H, Weissman DB (2022). Evolution of chromosome number in grasshoppers (Orthoptera: Caelifera: Acrididae). Org Divers Evol.

[B93] Ichikawa Y, Nishimura Y, Kurumizaka H, Shimizu M (2015). Nucleosome organization and chromatin dynamics in telomeres. Biomol Concepts.

[B94] Ijdo JW, Wells RA, Baldini A, Reeders ST (1991). Improved telomere detection using a telomere repeat probe (TTAGGG)n generated by PCR. Nucleic Acids Res.

[B95] Jetybayev IE, Bugrov AG, Karamysheva TV, Camacho JPM, Rubtsov NB (2012). Chromosomal localization of ribosomal and telomeric DNA provides new insights on the evolution of Gomphocerinae grasshoppers. Cytogenet Genome Res.

[B96] Jetybayev IY, Bugrov AG, Üna M, Buleu OG, Rubtsov NB (2017). Molecular cytogenetic analysis reveals the existence of two independent neo-XY sex chromosome systems in Anatolian Pamphagidae grasshoppers. BMC Evol Biol.

[B97] Kaiser VB, Bachtrog D (2010). Evolution of sex chromosomes in insects. Annu Rev Genet.

[B98] Kawagoshi T, Nishida C, Ota H, Kumazawa Y, Endo H, Matsuda Y (2008). Molecular structures of centromeric heterochromatin and karyotypic evolution in the Siamese crocodile (Crocodilus syamensis) (Crocodylidae, Crocodylia). Chromosome Res.

[B99] Kolquist KA, Ellisen LW, Counter CM, Meyerson M, Tan LK, Weinberg RA, Haber DA, Gerald WL (1998). Expression of TERT in early premalignant lesions and a subset of cells in normal tissues. Nat Genet.

[B100] Kong A, Thorleifsson G, Gudbjartsson DF, Masson G, Sigurdsson A, Jonasdottir A, Walters GB, Jonasdottir A, Gylfason A, Kristinsson KT (2010). Fine-scale recombination rate differences between sexes, populations and individuals. Nature Oct.

[B101] Kong CM, Lee XW, Wang X (2013). Telomere shortening in human diseases. FEBS J.

[B102] Kostmann A, Kratochvíl L, Rovatsos M (2020). First report of sex chromosomes in plated lizards (Squamata: Gerrhosauridae). Sex Dev.

[B103] Kramara J, Osia B, Malkova A (2018). Break-induced replication: The where, the why, and the how. Trends Genet.

[B104] Kretschmer R, Ferguson-Smith MA, Oliveira EHC (2018). Karyotype evolution in birds: from conventional staining to chromosome painting. Genes.

[B105] Kuznetsova V, Grozeva S, Gokhman V (2020). Telomere structure in insects: A review. J Zool Syst Evol Res.

[B106] Lanzone C, Labaroni C, Suárez N, Rodríguez D, Herrera ML, Bolzán AD (2015). Distribution of telomeric sequences (TTAGGG)n in rearranged chromosomes of phyllotine rodents (Cricetidae, sigmodontinae). Cytogenet Genome Res.

[B107] Lazzerini-Denchi E, Sfeir A (2016). Stop pulling my strings - what telomeres taught us about the DNA damage response. Nat Rev Mol Cell Biol.

[B108] Lee C, Sasi R, Lin CC (1993). Interstitial localization of telomeric DNA sequences in the Indian muntjac chromosomes: further evidence for tandem chromosome fusions in the karyotypic evolution of the Asian muntjacs. Cytogenet Cell Genet.

[B109] Lejnine S, Markov VL, Langmore JP (1995). Proc Natl Acad Sci USA.

[B110] Libertini A, Vitturi R, Lannino A, Maone MC, Franzoi P, Riccato F, Colomba S (2006). Fish mapping of 18S rDNA and (TTAGGG)n sequences in two pipefish species (Gasteroisteiformes: Syngnathidae). J Genet.

[B111] Lin KW, Yan J (2008). Endings in the middle: Current knowledge of interstitial telomeric sequences. Mutat Res.

[B112] Liu WS, Fredga K (1999). Telomeric (TTAGGG)n sequences are associated with nucleolus organizer regions (NORs) in the wood lemming. Chromosome Res.

[B113] Lopes J, Piazza A, Bermejo R, Kriegsman B, Colosio A, Teulade-Fichou MP, Foiani M, Nicolas A (2011). G-quadruplex-induced instability during leading-strand replication. EMBO J.

[B114] López-Fernández C, Pradillo E, Zabal-Aguirre M, Fernández JL, Garcia de la Vega C, Gosálvez J (2004). Telomeric and interstitial telomeric-like DNA sequence in Orthoptera genomes. Genome.

[B115] Luderus MEE, van Steensel B, Chong L, Sibon OCM, Cremers FFM, de Lange T (1996). Structure, subnuclear distribution, and nuclear matrix association of the mammalian telomeric complex. J Cell Biol.

[B116] Lukhtanov VA (2022). Diversity and evolution of telomere and subtelomere DNA sequences in insects. BioRxiv.

[B117] Machado CRD, Domit C, Pucci MB, Gazolla CB, Glugoski L, Nogaroto V, Vicari MR (2020). Heterochromatin and microsatellites detection in karyotypes of four sea turtle species: Interspecific chromosomal differences. Genet Mol Biol.

[B118] Machado CRD, Glugoski L, Domit C, Pucci MB, Goldberg DW, Marinho LA, Costa GWWF, Nogaroto V, Vicari MR (2020). Comparative cytogenetics of four sea turtle species (Cheloniidae): G-banding pattern and in situ localization of repetitive DNA units. Cytogenet Genome Res.

[B119] Maicher A, Lockhart A, Luke B (2014). Breaking new ground: Digging into TERRA function. Biochim Biophys Acta.

[B120] Makarov VL, Hirose Y, Langmore JP (1997). Long G tails at both ends of human chromosomes suggest a C strand degradation mechanism for telomere shortening. Cell.

[B121] Mason JM, Biessmann H (1995). The unusual telomeres of Drosophila. Trends Genet.

[B122] Mason JM, Frydrychova RC, Biessmann H (2008). Drosophila telomeres: an exception providing new insights. Bioessays.

[B123] Mason JM, Randall TA, Frydrychova RC (2016). Telomerase lost?. Chromosoma.

[B124] Matoso Silva R, Adega F, Kjöllerström HJ, Labuschagne K, Kotze A, Fernandes C, Chaves R, Oom MM (2016). Classical and molecular cytogenetics of the Panther Genet Genetta maculata (Mammalia, Carnivora, Viverridae). Cytogenet Genome Res.

[B125] Mattos TL, Coelho AC, Schneider CH, Telles DOC, Menin M, Gross MC (2014). Karyotypic diversity in seven Amazonian anurans in the genus Hypsiboas (family Hylidae). BMC Genetics.

[B126] Matzenbacher CA, Silva J, Garcia ALH, Kretschmer R, Cappetta M, Oliveira EHC, Freitas TRO (2022). Using telomeric length measurements and methylation to understand the karyotype diversification of Ctenomys Minutus (a small fossorial mammals). Genome.

[B127] Mazzoleni S, Augstenová B, Clemente L, Auer M, Fritz U, Praschag P, Protiva T, Velenský P, Kratochvíl L, Rovatsos M (2020). Sex is determined by XX/XY sex chromosomes in Australasian side-necked turtles (Testudines: Chelidae). Sci Rep.

[B128] Mazzoleni S, Schillaci O, Sineo L, Dumas F (2017). Distribution of interstitial telomeric sequences in primates and the pygmy tree shrew (Scandentia). Cytogenet Genome Res.

[B129] McClintock B (1941). The stability of broken ends of chromosomes in Zea mays. Genetics.

[B130] McClintock B, Moore JA (1987). The discovery and characterization of transposable elements.

[B131] Melek M, Shippen DE (1996). Chromosome healing: spontaneous and programmed de novo telomere formation by telomerase. BioEssays.

[B132] Mendoza O, Bourdoncle A, Boulé JB, Brosh RM, Mergny JL (2016). G-quadruplexes and helicases. Nucleic Acids Res.

[B133] Metcalfe CJ, Eldridge MDB, Toder R, Johnston PG (1998). Mapping the distribution of the telomeric sequence (T2AG3)n in the Macropodoidea (Marsupialia), by fluorescence in situ hybridization. I. The swamp wallaby, Wallabia bicolor. Chromosome Res.

[B134] Metcalfe CJ, Eldridge MDB, Johnston PG (2002). Mapping the distribution of the telomeric sequence (T2AG3)n in rock-wallabies, Petrogale (Marsupialia: Macropodidae), by fluorescence in situ hybridization. II. The lateralis complex. Cytogenet Genome Res.

[B135] Metcalfe CJ, Eldridge MDB, Johnston PG (2004). Mapping the distribution of the telomeric sequence (T2AG3)n in the 2n = 14 presumed ancestral marsupial complement and in the macropodine (Marsupialia: Macropodidae), by fluorescence in situ hybridization. Chromosome Res.

[B136] Metcalfe CJ, Eldridge MDB, Johnston PG (2007). Mapping the distribution of the telomeric sequence (T2AG3)n in the Macropodoidea (Marsupialia) by fluorescence in situ hybridization. II. The ancestral 2n = 22 macropodid karyotype. Cytogenet Genome Res.

[B137] Meyne J, Baker RJ, Hobart HH, Hsu TC, Ryder OA, Ward OG, Wiley JE, Wurster-Hill DH, Yates TL, Moyzis RK (1990). Distribution of non-telomeric sites of the (TTAGGG)n telomeric sequence in vertebrate chromosomes. Chromosoma.

[B138] Mignon-Ravix C, Depetris D, Delobel B, Croquette MF, Mattei MG (2002). A human interstitial telomere associates in vivo with specific TRF2 and TIN2 proteins. Eur J Hum Genet.

[B139] Milhomem SR, Pieczarka JC, Crampton WGR, Silva DS, De Souza ACP, Carvalho JR, Nagamachi CY (2008). Chromosomal evidence for a putative cryptic species in the Gymnotus carapo species-complex (Gymnotiformes, Gymnotidae). BMC Genet.

[B140] Mohan KN, Rani BS, Kulashreshta PS, Kadandale JS (2011). Characterization of TTAGG telomeric repeats, their interstitial occurrence and constitutively active telomerase in the mealybug Planococcus lilacinus (Homoptera; Coccoidea). Chromosoma.

[B141] Mondello C, Pirzio L, Azzalin CM, Giulotto E (2000). Instability of interstitial telomeric sequences in the human genome. Genomics.

[B142] Monti V, Serafini K, Manicardi GC, Mandrioli M (2013). Characterization of non-LTR retrotransposable TRAS elements in the aphids Acyrthosiphon pisum and Myzus persicae (Aphididae, Hemiptera). J Hered.

[B143] Mota-Velasco JC, Alves Ferreira J, Cioffi MB, Ocalewicz K, Campos-Ramos R, Shirak A, Lee BY, Martins C, Penman DJ (2010). Characterization of the chromosome fusions in Oreochromis karongae. Chromosome Res.

[B144] Mravinac B, Meštrović N, Čavrak V, Plohl M (2011). TCAGG, an alternative telomeric sequence in insects. Chromosoma.

[B145] Mudry MD, Nieves M, Bolzán AD (2007). Chromosomal localization of the telomeric (TTAGGG)n sequence in eight species of New World Primates (Neotropical Primates, Platyrrhini). Cytogenet Genome Res.

[B146] Muntoni A, Reddel RR (2005). The first molecular details of ALT in human tumor cells. Hum Mol Genet.

[B147] Murakami T, Maki N, Nishida-Umehara C, Matsuda Y, Agata K (2007). Establishment of high-resolution FISH mapping system and its application for molecular cytogenetic characterization of chromosomes in the newt Cynops pyrrhogaster (Urodela, Amphibia). Chromosome Res.

[B148] Nagamachi CY, Pieczarka JC, O’Brien PCM, Pinto JA, Malcher SM, Pereira AL, Rissino JD, Mendes-Oliveira AC, Rossi RV, Ferguson-Smith MA (2013). FISH with whole chromosome and telomeric probes demonstrates huge karyotypic reorganization with ITS between two species of Oryzomyini (Sigmodontinae, Rodentia): Hylaeamys megacephalus probes on Cerradomys langguthi karyotype. Chromosome Res.

[B149] Nanda I, Schrama D, Feichtinger W, Haaf T, Schartl M, Schmid M (2002). Distribution of telomeric (TTAGGG)n sequences in avian chromosomes. Chromosoma.

[B150] Nanda I, Karl E, Griffin DK, Schartl M, Schmid M (2007). Chromosome repatterning in three representative parrots (Psittaciformes) inferred from comparative chromosome painting. Cytogenet Genome Res.

[B151] Nergadze SG, Rocchi M, Azzalin CM, Mondello C, Giulotto E (2004). Insertion of telomeric repeats at intrachromosomal break sites during primate evolution. Genome Res.

[B152] Nielsen L, Edstrom JE (1993). Complex telomere-associated repeat units in members of the genus Chironomus evolve from sequences similar to simple telomeric repeats. Mol Cell Biol.

[B153] Nishida C, Ishijima J, Kosaka A, Tanabe H, Habermann FA, Griffin DK, Matsuda Y (2008). Characterization of chromosome structures of Falconinae (Falconidae, Falconiformes, Aves) by chromosome painting and delineation of chromosome rearrangements during their differentiation. Chromosome Res.

[B154] Nishida C, Ishijima J, Ishishita S, Yamada K, Griffin DK, Yamazaki T, Matsuda Y (2013). Karyotype reorganization with conserved genomic compartmentalization in dot-shaped microchromosomes in the japanese mountain hawk-eagle (Nisaetus nipalensis orientalis, Accipitridae). Cytogenet Genome Res.

[B155] O’Sullivan RJ, Karlseder J (2010). Telomeres: protecting chromosomes against genome instability. Nat Rev Mol Cell Biol.

[B156] Ocalewicz K (2013). Telomeres in Fishes. Cytogenet Genome Res.

[B157] Ocalewicz K, Śliwińska A, Jankun M (2004). Autosomal localization of internal telomeric sites (ITS) in brook trout, Salvelinus fontinalis (Pisces, Salmonidae). Cytogenet Genome Res.

[B158] Ocalewicz K, Mota-Velasco JC, Campos-Ramos R, Penman DJ (2009). FISH and DAPI staining of the synaptonemal complex of the Nile tilapia (Oreochromis niloticus) allow orientation of the unpaired region of bivalent 1 observed during early pachytene. Chromosome Res.

[B159] Oliveira MLM, Paim FG, Freitas ÉAS, Oliveira C, Foresti F (2021). Cytomolecular investigations using repetitive DNA probes contribute to the identification and characterization of Characidium sp. aff. C. vidali (Teleostei: Characiformes). Neotrop Ichthyol.

[B160] Oliveira VCS, Viana PF, Gross MC, Feldberg E, Da Silveira R, Cioffi MB, Bertollo LAC, Schneider CH (2021). Looking for genetic effects of polluted anthropized environments on Caiman crocodilus crocodilus (Reptilia, Crocodylia): A comparative genotoxic and chromosomal analysis. Ecotoxicol Environ Saf.

[B161] Olovnikov AM (1973). A theory of marginotomy. The incomplete copying of template margin in enzymic synthesis of polynucleotides and biological significance of the phenomenon. J Theor Biol.

[B162] Ono T, Yoshida MC (1997). Differences in the chromosomal distribution of telomeric (TTAGGG)n sequences in two species of the vespertilionid bats. Chromosome Res.

[B163] Osanai M, Kojima KK, Futahashi R, Yaguchi S, Fujiwara H (2006). Identification and characterization of the telomerase reverse transcriptase of Bombyx mori (silkworm) and Tribolium castaneum (flour beetle). Gene.

[B164] Paço A, Chaves R, Vieira-da-Silva A, Adega F (2013). The involvement of repetitive sequences in the remodelling of karyotypes: the Phodopus genomes (Rodentia, Cricetidae). Micron.

[B165] Paeschke K, Capra J, Zakian V (2011). DNA Replication through G-quadruplex motifs is promoted by the Saccharomyces cerevisiae Pif1 DNA helicase. Cell.

[B166] Palacios-Gimenez OM, Castillo ER, Martí DA, Cabral-de-Mello DC (2013). Tracking the evolution of sex chromosome systems in Melanoplinae grasshoppers through chromosomal mapping of repetitive DNA sequences. BMC Evol Biol.

[B167] Palacios-Gimenez OM, Carvalho CR, Soares FAF, Cabral-de-Mello DC (2015). Contrasting the chromosomal organization of repetitive DNAs in two Gryllidae crickets with highly divergent karyotypes. PloS One.

[B168] Palacios-Gimenez OM, Marti DA, Cabral-de-Mello DC (2015). Neo-sex chromosomes of Ronderosia bergi: insight into the evolution of sex chromosomes in grasshoppers. Chromosoma.

[B169] Pardue ML, DeBaryshe PG (2003). Retrotransposons provide an evolutionarily robust non-telomerase mechanism to maintain telomeres. Ann Rev Genet.

[B170] Parks MM, Lawrence CE, Raphael BJ (2015). Detecting non-allelic homologous recombination from high-throughput sequencing data. Genome Biol.

[B171] Perry J, Slater HR, Choo KH (2004). Centric fission - simple and complex mechanisms. Chromosome Res.

[B172] Pich U, Fuchs J, Schubert I (1996). How do Alliaceae stabilize their chromosome ends in the absence of TTAGGG sequences?. Chromosome Res.

[B173] Pierce AJ, Hu P, Han M, Ellis N, Jasin M (2001). Ku DNA end-binding protein modulates homologous repair of double-strand breaks in mammalian cells. Genes Dev.

[B174] Pisano S, Galati A, Cacchione S (2008). Telomeric nucleosomes: forgotten players at chromosome ends. Cell Mol Life Sci.

[B175] Pisano S, Leoni D, Galati A, Rhodes D, Savino M, Cacchione S (2010). The human telomeric protein hTRF1 induces telomere-specific nucleosome mobility. Nucleic Acids Res.

[B176] Pomianowski L, Jankun M, Ocalewicz K (2012). Detection of interstitial telomeric sequences in the Arctic charr (Salvelinus alpinus, Linnaeus 1758) (Teleostei, Salmonidae). Genome.

[B177] Poole LA, Cortez D (2016). SMARCAL1 and telomeres: Replicating the troublesome ends. Nucleus.

[B178] Prušáková D, Peska V, Pekár S, Bubeník M, Čížek L, Bezděk A, Frydrychová RC (2021). Telomeric DNA sequences in beetle taxa vary with species richness. Sci Rep.

[B179] Qi H, Zakian VA (2000). The Saccharomyces telomere-binding protein Cdc13p interacts with both the catalytic subunit of DNA polymerase alpha and the telomerase-associated est1 protein. Genes Dev.

[B180] Reed K, Phillips RB (1995). Molecular cytogenetic analysis of the double-CMA3 chromosome of lake trout, Salvelinus namaycush. Cytogenet Cell Genet.

[B181] Rego A, Marec F (2003). Telomeric and interstitial telomeric sequences in holokinetic chromosomes of Lepidoptera: Telomeric DNA mediates association between postpachytene bivalents in achiasmatic meiosis of females. Chromosome Res.

[B182] Rice C, Skordalakes E (2016). Structure and function of the telomeric CST complex. Comput Struct Biotechnol J.

[B183] Rivero MT, Mosquera A, Goyanes V, Slijepcevic P, Fernandez JL (2004). Differences in repair profiles of interstitial telomeric sites between normal and DNA double-strand break repair deficient Chinese hamster cells. Exp Cell Res.

[B184] Robin JD, Magdinier F (2016). Physiological and pathological aging affects chromatin dynamics, structure and function at the nuclear edge. Front Genet.

[B185] Robin JD, Ludlow AT, Batten K, Magdinier F, Stadler G, Wagner KR, Shay JW, Wright WE (2014). Telomere position effect: Regulation of gene expression with progressive telomere shortening over long distances. Genes Dev.

[B186] Robin JD, Ludlow AT, Batten K, Gaillard MC, Stadler G, Magdinier F, Wright WE, Shay JW (2015). SORBS2 transcription is activated by telomere position effect-over long distance upon telomere shortening in muscle cells from patients with facioscapulohumeral dystrophy. Genome Res.

[B187] Rocco L, Costagliola D, Stingo V (2001). (TTAGGG)n telomeric sequence in selachian chromosomes. Heredity (Edinb).

[B188] Rocco L, Morescalchi MA, Costagliola D, Stingo V (2002). Karyotype and genome characterization in four cartilaginous fishes. Gene.

[B189] Rodgers K, McVey M (2016). Error-prone repair of DNA double-strand breaks. J Cell Physiol.

[B190] Rodrigues BS, Kretschmer R, Gunski RJ, Garnero ADV, O’Brien PCM, Ferguson-Smith MA, de Oliveira EHC (2017). Chromosome painting in tyrant flycatchers confirms a set of inversions shared by Oscines and Suboscines (Aves, Passeriformes). Cytogenet Genome Res.

[B191] Rosa KO, Ziemniczak K, Barros AV, Nogaroto V, Almeida MC, Cestari MM, Artoni RF, Vicari MR (2012). Numeric and structural chromosome polymorphism in Rineloricaria lima (Siluriformes: Loricariidae): fusion points carrying 5S rDNA or telomere sequence vestiges. Rev Fish Biol Fish.

[B192] Rossi AR, Gornung E, Sola L, Nirchio M (2005). Comparative molecular cytogenetic analysis of two congeneric species, Mugil curema and M. liza (Pisces, Mugiliformes), characterized by significant karyotype diversity. Genetica.

[B193] Rovatsos MT, Marchal JA, Romero-Fernández I, Fernández FJ, Giagia-Athanosopoulou EB, Sánchez A (2011). Rapid, independent, and extensive amplification of telomeric repeats in pericentromeric regions in karyotypes of arvicoline rodents. Chromosome Res.

[B194] Rovatsos M, Kratochvíl L, Altmanová M, Johnson Pokorná M (2015). Interstitial telomeric motifs in squamate reptiles: when the exceptions outnumber the rule. PloS One.

[B195] Ruiz-Herrera A, García F, Azzalin C, Giulotto E, Egozcue J, Ponsà M, Garcia M (2002). Distribution of intrachromosomal telomeric sequences (ITS) on Macaca fascicularis (Primates) chromosomes and their implication for chromosome evolution. Hum Genet.

[B196] Ruiz-Herrera A, García F, Giulotto E, Attolini C, Egozcue J, Ponsà M, Garcia M (2005). Evolutionary breakpoints are co-localized with fragile sites and intrachromosomal telomeric sequences in primates. Cytogenet Genome Res.

[B197] Ruiz-Herrera A, Nergadze SG, Santagostino M, Giulotto E (2008). Telomeric repeats far from the ends: mechanisms of origin and role in Evolution. Cytogenet Genome Res.

[B198] Salvadori S, Deiana A, Coluccia E, Florida G, Rossi E, Zuffardi O (1995). Colocalization of (TTAGGG)n telomeric sequences and ribosomal genes in Atlantic eels. Chromosome Res.

[B199] Salvati E, Scarsella M, Porru M, Rizzo A, Iachettini S, Tentori L, Graziani G, D’Incalci M, Stevens MF, Orlandi A (2010). PARP1 is activated at telomeres upon G4 stabilization: possible target for telomere-based therapy. Oncogene.

[B200] Santos-Pereira JM, Aguilera A (2015). R loops: New modulators of genome dynamics and function. Nat Rev Genet.

[B201] Sassi FMC, Deon GA, Moreira-Filho O, Vicari MR, Bertollo LAC, Liehr T, de Oliveira EA, Cioffi MB (2020). Multiple sex chromosomes and evolutionary relationships in Amazonian Catfishes: The outstanding model of the genus Harttia (Siluriformes: Loricariidae). Genes.

[B202] Scacchetti PC, Pansonato-Alves JC, Utsonomia R, Oliveira C, Foresti F (2011). Karyotypic diversity in four species of the genus Gymnotus Linnaeus, 1758 (Teleostei, Gymnotiformes, Gymnotidae): physical mapping of ribosomal genes and telomeric sequences. Comp Cytogenet.

[B203] Scacchetti PC, Utsunomia R, Pansonato-Alves JC, da Costa Silva GJ, Vicari MR, Artoni RF, Oliveira C, Foresti F (2015). Repetitive DNA sequences and evolution of ZZ/ZW sex chromosomes in Characidium (Teleostei: Characiformes). PloS One.

[B204] Scherthan H, Brandham PE, Bennet MD (1995). Kew Chromosome Conference IV.

[B205] Schmid M, Steinlein C (2016). Chromosome Banding in Amphibia. XXXIV. Intrachromosomal telomeric DNA sequences in Anura. Cytogenet Genome Res.

[B206] Schmid M, Steinlein C, Haaf T, Feightinger W, Guttenbach M, Bogart JP, Gruber SL, Kasahara S, Kakampuy W, Del Pino E (2018). The Arboranan frogs: evolution, biology, and cytogenetics. Cytogenet Genome Res.

[B207] Sember A, Bohlen J, Slechtová V, Altmanová M, Symonová R, Ráb P (2015). Karyotype differentiation in 19 species of river loach fishes (Nemacheilidae, Teleostei): extensive variability associated with rDNA and heterochromatin distribution and its phylogenetic and ecological interpretation. BMC Evol Biol.

[B208] Sember A, Bohlen J, Slechtová V, Altmanová M, Pelikánová S, Ráb P (2018). Dynamics of tandemly repeated DNA sequences during evolution of diploid and tetraploid botiid loaches (Teleostei: Cobitoidea: Botiidae). PloS One.

[B209] Seol JH, Shim EY, Lee SE (2018). Microhomology-mediated end joining: Good, bad and ugly. Mutat Res Mol Mech Mutagen.

[B210] Sfeir A, Kosiyatrakul ST, Hockemeyer D, MacRae SL, Karlseder J, Schildkraut CL, de Lange T (2009). Mammalian telomeres resemble fragile sites and require TRF1 for efficient replication. Cell.

[B211] Silva JB, Suárez P, Nagamachi CY, Carter TF, Pieczarka JC (2014). Cytogenetics of the Brazilian Bolitoglossa paraensis (Unterstein, 1930) salamanders (Caudata, Plethodontidae). Genet Mol Biol.

[B212] Simonet T, Zaragosi LE, Philippe C, Lebrigand K, Schouteden C, Augereau A, Bauwens S, Ye J, Santagostino M, Giulotto E (2011). The human TTAGGG repeat factors 1 and 2 bind to a subset of interstitial telomeric sequences and satellite repeats. Cell Res.

[B213] Slijepcevic P (1998). Telomeres and mechanisms of Robertsonian fusion. Chromosoma.

[B214] Slijepcevic P (2016). Mechanisms of the evolutionary chromosome plasticity: integrating the “Centromere-from-Telomere” hypothesis with telomere length regulation. Cytogenet Genome Res.

[B215] Slijepcevic P, Al-Wahiby S (2005). Telomere biology: integrating chromosomal end protection with DNA damage response. Chromosoma.

[B216] Slijepcevic P, Xiao Y, Natarajan AT, Bryant PE (1997). Instability of CHO chromosomes containing interstitial telomeric sequences originating from Chinese hamster chromosome 10. Cytogenet Cell Genet.

[B217] Smith JS, Chen Q, Yatsunyk LA, Nicoludis JM, Garcia MS, Kranaster R, Balasubramanian S, Monchaud D, Teulade-Fichou MP, Abramowitz L (2011). Rudimentary G-quadruplex-based telomere capping in Saccharomyces cerevisiae. Nat Struct Mol Biol.

[B218] Suárez P, Cardozo D, Baldo D, Pereyra MO, Faivovich J, Orrico VGD, Castroli GF, Grabiele M, Bernarde PS, Nagamachi CY (2013). Chromosome evolution in Dendropsophini (Amphibia, Anura, Hylinae). Cytogenet Genome Res.

[B219] Suárez P, Ferro JM, Nagamachi CY, Cardozo DE, Blasco-Zúñiga A, Silva JB, Marciano-Jr E, Costa MA, Orrico VGD, Solé M (2020). Chromosome evolution in Lophyohylini (Amphibia, Anura, Hylinae). PloS One.

[B220] Sundquist WI, Klug A (1989). Telomeric DNA dimerizes by formation of guanine tetrads between hairpin loops. Nature.

[B221] Swier VJ, Khan FAA, Baker RJ (2012). Do time, heterochromatin, NORs, or chromosomal rearrangements correlate with distribution of interstitial telomeric repeats in Sigmodon (cotton rats)?. J Hered.

[B222] Teixeira LSR, Seger KR, Targueta CP, Orrico VGD, Lourenço LB (2016). Comparative cytogenetics of tree frogs of the Dendropsophus marmoratus (Laurenti, 1768) group: conserved karyotypes and interstitial telomeric sequences. Comp Cytogenet.

[B223] Tomaska L, Nosek J (2009). Telomere heterogeneity: taking advantage of stochastic events. FEBS Lett.

[B224] Tommerup H, Dousmanis A, de Lange T (1994). Unusual chromatin in human telomeres. Mol Cell Biol.

[B225] van Steensel B, Smogorzewska A, de Lange T (1998). TRF2 protects human telomeres from end-to-end fusions. Cell.

[B226] Vannier JB, Pavicic-Kaltenbrunner V, Petalcorin MI, Ding H, Boulton SJ (2012). RTEL1 dismantles T loops and counteracts telomeric G4-DNA to maintain telomere integrity. Cell.

[B227] Vannier JB, Sarek G, Boulton SJ (2014). RTEL1: Functions of a disease-associated helicase. Trends Cell Biol.

[B228] Ventura K, Silva MJ, Fagundes V, Christoff AU, Yonenaga-Yassuda Y (2006). Non-telomeric sites as evidence of chromosomal rearrangement and repetitive (TTAGGG)n arrays in heterochromatic and euchromatic regions in four species of Akodon (Rodentia, Muridae). Cytogenet Genome Res.

[B229] Ventura M, Catacchio CR, Sajjadian S, Vives L, Sudmant PH, Marques-Bonet T, Graves TA, Wilson RK, Eichler EE (2012). The evolution of African great ape subtelomeric heterochromatin and the fusion of human chromosome 2. Genome Res.

[B230] Vermeesch JR, De Meurichy W, Van Den Berghe H, Marynen P, Petit P (1996). Differences in the distribution and nature of the interstitial telomeric (TTAGGG)n sequences in the chromosomes of the Giraffidae, okapi (Okapia johnstoni), and giraffe (Giraffa camelopardalis): evidence for ancestral telomeres at the okapi polymorphic rob(5; 26) fusion site. Cytogenet Cell Genet.

[B231] Viana PF, Ribeiro LB, Souza GM, Chalkidis HM, Gross MC, Feldberg E (2016). Is the karyotype of neotropical boid snakes really conserved? Cytotaxonomy, chromosomal rearrangements and karyotype organization in the Boidae family. PloS One.

[B232] Vítková M, Král J, Traut W, Zrzavý J, Marec F (2005). The evolutionary origin of insect telomeric repeats, (TTAGG)n. Chromosome Res.

[B233] Warchałowska-Śliwa E, Grzywacz B, Kociński M, Maryańska-Nadachowska A, Heller K-G, Hemp C (2021). Highly divergent karyotypes and barcoding of the East African genus Gonatoxia Karsch (Orthoptera: Phaneropterinae). Sci Rep.

[B234] Watson JD (1972). Origin of concatemeric T7 DNA. Nature New Biol.

[B235] Wijayanto H, Hirai Y, Kamanaka Y, Katho A, Sajuthi D, Hirai H (2005). Patterns of C-heterochromatin and telomeric DNA in two representative groups of small apes, the genera Hylobates and Symphalangus. Chromosome Res.

[B236] Wiley JE, Meyne J, Little M, Stout JC (1992). Interstitial hybridization sites of the (TTTGGG)n telomeric sequence on the chromosomes of some North American hylid frogs. Cytogenet Cell Genet.

[B237] Williamson JR, Raghuraman MKK, Cech TR (1989). Monovalent cation-induced structure of telomeric DNA: The G-quartet model. Cell.

[B238] Wood AM, Rendtlew Danielsen JM, Lucas CA, Rice EL, Scalzo D, Shimi T, Goldman RD, Smith ED, Le Beau MM, Kosak ST (2014). TRF2 and lamin A/C interact to facilitate the functional organization of chromosome ends. Nat Commun.

[B239] Wood AM, Laster K, Rice EL, Kosak ST (2015). A beginning of the end: new insights into the functional organization of telomeres. Nucleus.

[B240] Wu P, van Overbeek M, Rooney S, de Lange T (2010). Apollo contributes to G overhang maintenance and protects leading-end telomeres. Mol Cell.

[B241] Xu Y, Suzuki Y, Ito K, Komiyama M (2010). Telomeric repeat-containing RNA structure in living cells. Proc Natl Acad Sci U S A.

[B242] Yang D, Xiong Y, Kim H, He Q, Li Y, Chen R, Songyang Z (2011). Human telomeric proteins occupy selective interstitial sites. Cell Res.

[B243] Zahler AM, Williamson JR, Cech TR, Prescott DM (1991). Inhibition of telomerase by G-quartet DMA structures. Nature.

[B244] Zakian VA (1995). Telomeres: beginning to understand the end. Science.

[B245] Zattera ML, Lima L, Duarte I, Sousa D, Araujo O, Gazoni T, Motti T, Recco-Pimental SM, Bruschi DP (2019). Chromosome spreading of the (TTAGGG)n repeats in the Pipa carvalhoi Miranda-Ribeiro, 1937 (Pipidae, Anura) karyotype. Comp Cytogenet.

[B246] Zou Y, Yi X, Wright WE, Shay JW (2002). Human telomerase can immortalize Indian muntjac cells. Exp Cell Res.

