# Supplementary material for: Telomere organization and the interstitial telomeric sites involvement in insects and vertebrates chromosome evolution
Source: Genet Mol Biol. 2022 Nov 14;45(3 Suppl 1):e20220071. doi: 10.1590/1678-4685-GMB-2022-0071 (PMC9693754; doi:10.1590/1678-4685-GMB-2022-0071)
Supplement: Supplementary Material S1 - [file 1415-4757-GMB-45-3-s1-e20220071-s1.pdf]

Genetics and Molecular Biology
DOI: https://doi.org/10.1590/1678-4685-GMB-2022-0071

Supplementary Material to "Telomere organization and the interstitial telomeric sites involvement in insects and vertebrates chromosome evolution"

Supplementary material S1 - Telomere probe amplification by PCR.

The general telomere sequence of vertebrates $(TTAGGG)_n$ can be amplified following Ijdo *et al.* (1991) in a PCR without DNA template. The PCR amplification consists of auto annealing of the primers telomere Fw 5'- $(TTAGGG)_5$ - 3' and telomere Rv 5' -$(CCCTAA)_5$ - 3'. The same strategy can be used in insects using the insect telomere motifs as primers. In addition to amplification, the telomere can be synthetized as a probe in PCR amplification using a nucleotide coupled to an hapten, usually digoxigenin 11-dUTP or biotin 16-dUTP. To obtain a general telomere vertebrate probe, perform the following reaction:

Test of amplification – unlabeled PCR

- 5 µL *Taq* DNA polymerase buffer (buffer 10×)
- 2,5 µL $MgCl_2$ (50 mM)
- 0,2 µL primer telomere Fw (100 µM)
- 0,2 µL primer telomere Rv (100 µM)
- 4 µL mix dNTPs (2 mM)
- 0,4 µL *Taq* DNA polymerase (5U/µL)
- 37,7 µL ultrapure H_2O
- total of the reaction = 50 µL.

Cycling

One step 94 °C for 5 min

10 cycles of (low stringency):

94 °C for 1 min

55 °C for 30 s

72 °C for 1 min

30 cycles of (high stringency):

94 °C for 1 min

60 °C for 30 s

72 °C for 1 min and 30 s

72 °C for 5 min

Note$_1$: Check for PCR products in 1% agarose gel. The PCR products should form a smear between 100 and 1,000 bp, then proceed to a labeled PCR. In the case of short PCR products, usually smaller than 200 bp, primer concentration in the reaction should be decreased. In opposite, if PCR products are largest than 1,000 bp, increase primers concentration in the PCR mixture to obtain a reasonable smear in agarose gel, i.e., 100 – 600 bp or 100 – 1,000 bp.

Note$_2$: The unlabeled PCR is used just for DNA fragments length adjustments.

Note$_3$: It is expected a smaller smear (DNA fragments length) after labeled PCR compared to unlabeled PCR, due to the use of a hapten nucleotide.

Labeled PCR

- 5 µL *Taq* DNA polymerase buffer (buffer 10×)
- 2,5 µL $MgCl_2$ (50 mM)
- 0,2 µL primer telomere Fw (100 µM)
- 0,2 µL primer telomere Rv (100 µM)
- 1 µL dATP (2 mM)
- 1 µL dCTP (2 mM)
- 1 µL dGTP (2 mM)
- 0,7 µL dTTP (2 mM)
- 0,6 µL digoxigenin 11-dUTP or another labeled nucleotide (1 mM)
- 0,4 µL *Taq* DNA polymerase (5U/µL)
- 37,4 µL ultrapure H_2O
- total of the reaction = 50 µL.

Note$_4$: Check for PCR products in 1% agarose gel. The PCR products should form a smear between 100 and 600 bp. If PCR products are in size length expected, proceed to FISH.
